# Study on SHP2 Conformational Transition and Structural Characterization of Its High-Potency Allosteric Inhibitors by Molecular Dynamics Simulations Combined with Machine Learning

**DOI:** 10.3390/molecules30010014

**Published:** 2024-12-24

**Authors:** Baerlike Wujieti, Mingtian Hao, Erxia Liu, Luqi Zhou, Huanchao Wang, Yu Zhang, Wei Cui, Bozhen Chen

**Affiliations:** School of Chemical Sciences, University of Chinese Academy of Sciences, No. 19A, Yuquan Road, Beijing 100049, China; wujietibaerlike21@mails.ucas.ac.cn (B.W.); haomingtian19@mails.ucas.ac.cn (M.H.); liuerxia21@mails.ucas.ac.cn (E.L.); zhouluqi22@mails.ucas.ac.cn (L.Z.); wanghuanchao22@mails.ucas.ac.cn (H.W.); zhangyu239@mails.ucas.ac.cn (Y.Z.)

**Keywords:** molecular docking, molecular dynamics simulations, MM/GBSA binding free energy calculations, enhanced sampling, machine learning

## Abstract

The src-homology 2 domain-containing phosphatase 2 (SHP2) is a human cytoplasmic protein tyrosine phosphatase that plays a crucial role in cellular signal transduction. Aberrant activation and mutations of SHP2 are associated with tumor growth and immune suppression, thus making it a potential target for cancer therapy. Initially, researchers sought to develop inhibitors targeting SHP2’s catalytic site (protein tyrosine phosphatase domain, PTP). Due to limitations such as conservativeness and poor membrane permeability, SHP2 was once considered a challenging drug target. Nevertheless, with the in-depth investigations into the conformational switch mechanism from SHP2’s inactive to active state and the emergence of various SHP2 allosteric inhibitors, new hope has been brought to this target. In this study, we investigated the interaction models of various allosteric inhibitors with SHP2 using molecular dynamics simulations. Meanwhile, we explored the free energy landscape of SHP2 activation using enhanced sampling technique (meta-dynamics simulations), which provides insights into its conformational changes and activation mechanism. Furthermore, to biophysically interpret high-dimensional simulation trajectories, we employed interpretable machine learning methods, specifically extreme gradient boosting (XGBoost) with Shapley additive explanations (SHAP), to comprehensively analyze the simulation data. This approach allowed us to identify and highlight key structural features driving SHP2 conformational dynamics and regulating the activity of the allosteric inhibitor. These studies not only enhance our understanding of SHP2’s conformational switch mechanism but also offer crucial insights for designing potent allosteric SHP2 inhibitors and addressing drug resistance issues.

## 1. Introduction

The src-homology 2 domain-containing phosphatase 2 (SHP2) is a human cytoplasmic protein tyrosine phosphatase (PTP) encoded by the PTPN11 gene, involved in regulating cell signaling pathways activated by cytokines, growth factors, and hormones [1,2,3]. Upregulation and mutations of SHP2 are associated with the development and progression of various human tumors, making it the only confirmed proto-oncoprotein in the PTP family. Specifically, aberrantly activated SHP2 interacts cooperatively with receptor tyrosine kinases (RTKs) and the KRAS signaling pathway, thereby promoting tumor cell growth [4]. It also serves as a downstream sensor in the PD-1 signaling pathway, capable of suppressing the immune response of T cells against tumors [5,6,7]. Consequently, SHP2 plays a crucial regulatory role in cancer cell proliferation and immune responses, thus making it an ideal target for cancer therapy, which has garnered significant attention from researchers in recent years.

**Figure 1:**
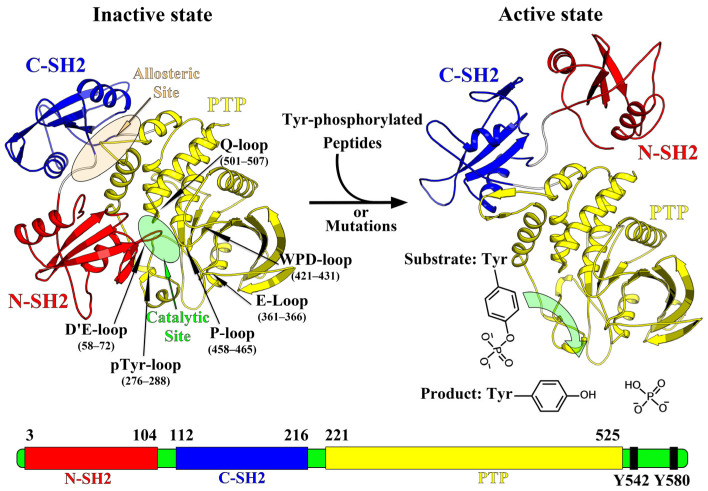
The overall structure of SHP2 and its allosteric regulatory mechanism.

The SHP2 includes two tandem SH2 domains (N-SH2 and C-SH2, residues 1–220), a catalytic protein tyrosine phosphatase domain (PTP, residues 221–525), and a poorly ordered C-terminal tail (residues 526–593) containing two phosphorylation sites (Tyr542 and Tyr580) (Figure 1) [1,2,3,8,9,10,11]. The PTP catalytic domain is composed of the P-loop (residues 458–465), pTyr recognition loop (pTyr loop, residues 276–288), WPD loop (residues 421–431), E-loop (residues 361–366), and Q-loop (residues 501–507) (Figure 1). Functionally, the P-loop, pTyr loop, and WPD loop are classified into fundamental loop regions, while the E-loop and Q-loop characterized by the significant glutamate and glutamine residues belong to assistant loop regions [3]. The fundamental loop regions cooperate to accommodate the acceptance and turnover of pTyr-containing substrate, while the assistant loop regions mainly assist in enhancing the substrate binding affinity and stabilizing the transition states of SHP2 during catalysis. Early on, researchers aimed to develop inhibitors targeting the catalytic site (PTP domain), such as compounds containing salicylic acid and sulfonic acid moieties [12,13], along with certain natural products. However, within the PTP family, the phosphatase catalytic domain is highly conserved, resulting in a lack of selectivity for inhibitors targeting the catalytic sites across highly homologous proteins such as SHP1, SHP2, and PTP1B [14]. Additionally, the polar and ionic functional groups in these inhibitors mimic the interactions between phosphorylated substrates and the catalytic center, leading to poor cellular permeability and bioavailability, which pose challenges for drug development.

In the basal state of SHP2, the N-SH2 domain binds to the PTP domain, adopting a closed self-inhibited conformation, devoid of phosphatase activity (Figure 1). Specifically, the D’E loop of the N-SH2 domain (comprising residues N58, T59, G60, D61, Y62, and A72) inserts into the catalytic cleft of the PTP domain (consisting of residues C459, D464, R465, Q506, and G503) to block substrate access to the catalytic active site and exert steric inhibition on phosphatase activity. When SHP2 is recruited by receptor tyrosine kinases (RTKs), either the binding of tyrosine-phosphorylated peptides or oncogenic mutations in PTPN11 disrupts the interface interaction between the N-SH2 and PTP domains. This results in the separation of the N-SH2 domain from the PTP domain, exposing the active site and activating the phosphatase activity of the PTP domain. For example, mutations such as the E76K mutation found in leukemia and certain solid tumors [15], the Q51E mutation leading to hypertrophic cardiomyopathy [16], and the common Q506P mutation in the PTP domain dramatically disrupt the interface interactions between the N-SH2 and PTP domains [17], resulting in the overactivation of SHP2. This allosteric regulation mechanism of SHP2 offers an opportunity for the development of allosteric inhibitors. Allosteric inhibitors achieve inhibition by stabilizing the closed self-inhibited conformation (inactive state) of SHP2. In 2015, Novartis reported that SHP099 [18], a novel allosteric phosphatase inhibitor, stabilized SHP2 in an autoinhibited closed conformation (inactive state). The binding site is a tunnel at the interface of the C-SH2 and PTP domain (Figure 1). Since then, several series of allosteric SHP2 inhibitors, bearing diverse chemical scaffolds, have been disclosed, including pyrazines, pyrimidinones, and various types of 5,6-fused bicyclic systems, namely, pyrazolopyrimidinones and pyrazolopyrazines [1]. To date, eight allosteric inhibitors of SHP2, including JAB-3068 [1], JAB-3312 [19], TNO155 [20], RMC-4630 [21], BBP-398 [22], ERAS-601 [23], RLY-1971 [24], and SH3809 [2], have entered clinical trials for cancer therapy.

Understanding the activation mechanism of SHP2 is crucial for the development of highly effective inhibitors. To achieve this goal, multiple research groups have resolved the atomic-level structural details of SHP2 in active, inactive, and inhibitor-bound states using crystallographic methods. However, these static structures are insufficient to determine intermediate states in the active/inactive state transition processes, as well as potential residues and interactions that control the protein conformational changes. Molecular dynamics (MD) simulations reveal the motion of atoms and molecules on time scales at high resolution, which is crucial for understanding the protein conformational changes. Nonetheless, MD simulations are typically performed at femtosecond resolution, resulting in the generation of extensive high-dimensional data, including atomic coordinates, velocities, forces, and energies. Extracting features related to protein dynamics and conformational changes from this vast dataset is a formidable task. Relying solely on the visualization of simulation trajectories often fails to fully exploit the potential information. To overcome this challenge, machine learning methods, particularly interpretable machine learning models such as the tree-based extreme gradient boosting (XGBoost) model [25] combined with the Shapley additive explanation (SHAP) method [26], can play a pivotal role in the analysis of simulation results [27,28,29,30,31]. The XGBoost model and SHAP method offer unique advantages in the analysis of molecular dynamics simulation data. They can handle complex protein structural dynamic information, aid in simplifying data, and reduce data dimensionality. Meanwhile, they can explain the impact of specific atoms or residues on the evolution of protein conformation in simulation trajectories.

In this study, we investigated the interaction model of 45 allosteric inhibitors (including 18 weakly active and 27 strongly active inhibitors) (Figure S1) with SHP2 by equilibrium molecular dynamics simulations and calculated the thermodynamic data of inhibitor binding by the Molecular Mechanics/Generalized Born Surface Area (MM/GBSA) method [32,33,34,35,36,37,38]. Meanwhile, we explored the free energy landscapes of apo-SHP2 (the unbound inhibitor state of SHP2) and five representative allosteric inhibitor-bound SHP2 (RMC-4550, IACS-13909, SHP389, SHP099, and Compound **7**) by meta-dynamics simulations (Meta-MD) [39]. These free energy landscapes not only addressed the possible stable, metastable, and transition states of SHP2, but also predicted the kinetic data of the SHP2 activation pathway. Most importantly, we extracted trajectory analysis data, ligand–receptor interaction fingerprints, and residue contact matrices as input conformational features from the above large-scale dynamics simulation trajectories and utilized an interpretable machine learning model, the extreme gradient boosting (XGBoost) model with the Shapley additive explanations (SHAP) method, to identify potential residues and interactions controlling the conformational changes of SHP2. This is of great significance for us in understanding the design principles of allosteric SHP2 inhibitors and avoiding drug resistance.

## 2. Results and Discussion

### 2.1. Interaction of Allosteric Inhibitors with SHP2

To analyze the interaction models of allosteric inhibitors with SHP2 and elucidate the structural requirements for achieving favorable biological activity of these inhibitors, we selected 45 allosteric inhibitor-bound SHP2 and apo-SHP2 (inactive state) to perform 100 ns equilibrium molecular dynamics simulations. After completing the simulations, the time-dependent energy (potential, kinetic, and total) curves of each simulated system, as well as the root mean square deviation (RMSD) curves of the SHP2 backbone atoms versus time, were calculated based on the MD simulation trajectories (Figures S2 and S3). Both the energy and RMSD curves of each simulated system reached satisfactory convergence at around 50 ns. In other words, the global energetic and structural properties of each MD simulation had stabilized. Therefore, it was reasonable to perform trajectory analysis and thermodynamic analysis based on the stable structures of each simulated system, which were taken between 50 and 100 ns.

**Figure 2:**
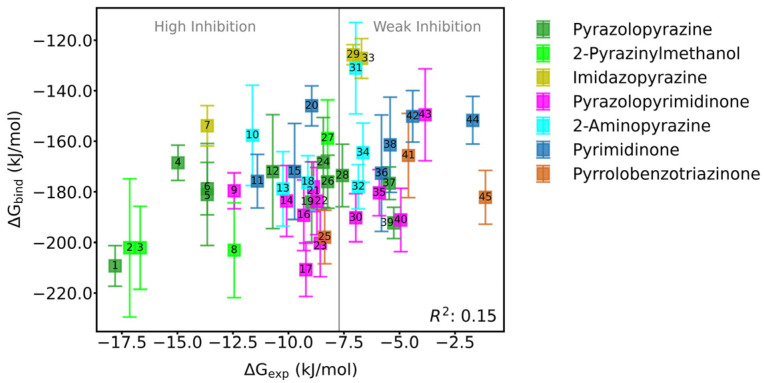
Scatter plot of binding free energy (ΔG_bind_) vs. experimental inhibitory activity (ΔG_exp_) for allosteric inhibitors to SHP2. The scatters were color-coded according to the mother nucleus of the allosteric inhibitors. The structural details were given in Figure S1.

All 45 allosteric inhibitors studied in the simulation bound to the “tunnel”-like pocket at the interface between the C-SH2 and PTP domains of SHP2, which is the allosteric site of SHP2 (Figure 1). To evaluate the binding affinity and stability of each allosteric inhibitor at SHP2’s allosteric site, we calculated the binding free energies (ΔG_bind_) of each inhibitor with SHP2 using the MM/GBSA method, based on the equilibrium segment trajectories (50~100 ns) from molecular dynamics simulations. As shown in Table S1 and Figure 2, the binding free energies of the 45 allosteric inhibitors with SHP2 range from −210.80 to −125.74 kJ/mol, indicating that both weakly and strongly active inhibitors have high binding affinity for SHP2’s allosteric site. From the binding energy subterms, we infer that the primary favorable contribution to inhibitor binding at SHP2’s allosteric site is the van der Waals term (ΔE_vdw_), followed by the electrostatic term (ΔE_ele_). In contrast, the polar solvation energy term (ΔG_GB_) is unfavorable for inhibitor binding at SHP2’s allosteric site. Furthermore, we attempted to establish a correlation between the binding free energy and experimental inhibitory activity (IC50 value was converted to ΔG_exp_ using the following relationship: ΔG_exp_ = −RT × ln(K_a_) = RT × ln(K_d_) = RT × ln(IC50), where K_a_ was the binding equilibrium constant, K_d_ was the dissociation equilibrium constant, R was the gas constant, and T was the temperature), but the correlation coefficient (R^2^) was only 0.15. Analyzing the reason for the weak correlation, one possibility is the error introduced by differences in experimental conditions and testing methods. Another possibility is that allosteric inhibitors are not competitive with endogenous ligands at the orthosteric site (the catalytically active site), and their inhibitory activity depends not only on the thermodynamic properties of binding but also on the kinetic properties driven by allosteric effects.

**Figure 3:**
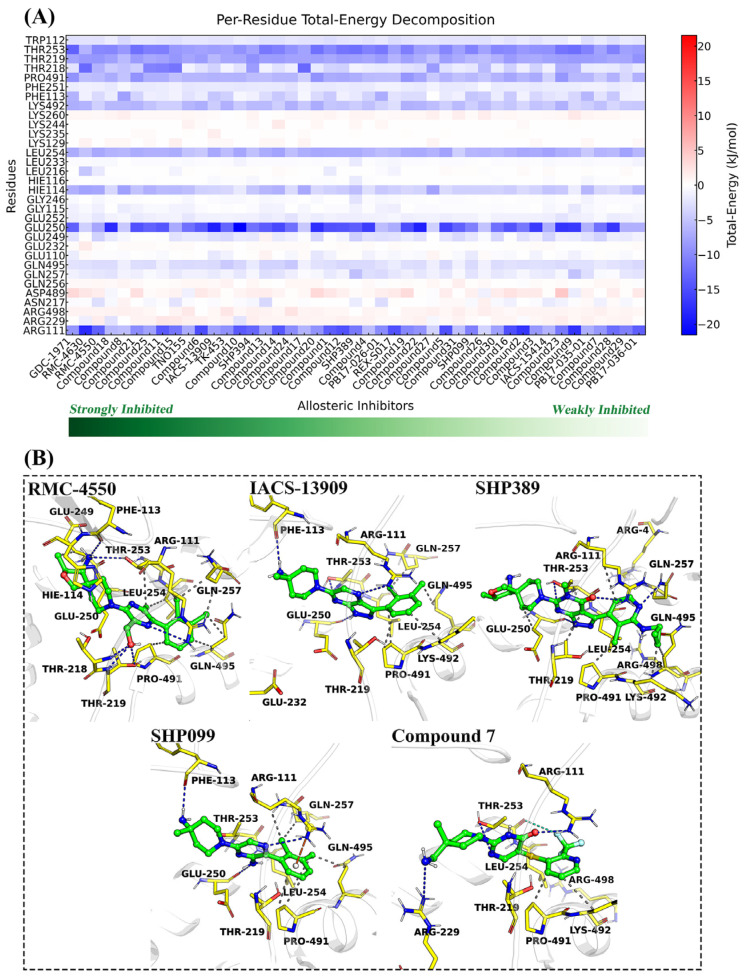
Interaction energies between allosteric inhibitors and SHP2 residues (**A**) and the interaction analysis of five representative allosteric inhibitors with SHP2 (**B**).

By decomposing the binding free energy into the interaction energies between the inhibitor and individual residues at SHP2’s allosteric site (Figure 3A), we identified key residues and interactions associated with SHP2’s allosteric effects. For most allosteric inhibitors, Arg111, Trp112, Phe113, Hie114 (His114), Thr218, Thr219, Glu250, Phe251, Glu252, Thr253, Leu254, Gln257, Pro491, Lys492, and Gln495 make significantly positive contributions (≤−1.26 kJ/mol) to the binding free energy of the inhibitors. These residues are not only important for the stable binding of allosteric inhibitors to SHP2 but are also directly involved in SHP2’s allosteric regulation. In contrast, Arg229, Glu232, Gln256, Lys260, Asp489, and Arg498 contribute negatively (≥1.26 kJ/mol) to the binding free energy of the inhibitors, suggesting that these residues are not conducive to the stable binding of allosteric inhibitors to SHP2 and thus represent potential optimization sites. Future development of allosteric inhibitors with novel structures should focus on improving interactions with these residues. Additionally, by analyzing the individual components of interaction energies separately (Figure S4), we found that Arg111, Phe113, and Glu250 make significantly positive contributions (≤−5.86 kJ/mol) to electrostatic interaction energies, indicating that these three residues can form either hydrogen bonds or electrostatic interactions with allosteric inhibitors. Arg111, Hie114, Thr218, Thr219, Glu250, Thr253, Leu254, Pro491, and Lys492 also contribute positively (≤−2.09 kJ/mol) to van der Waals interaction energies, indicating that these nine residues can form hydrophobic interactions with allosteric inhibitors.

To further analyze the interaction model between the inhibitor and residues at SHP2’s allosteric site, high-frequency interaction fingerprints (frequencies > 0.40) were determined based on the equilibrium segment trajectories using the PLIP software 1.1.0 (Figure 3B and Figure S5). The specific interactions are summarized as follows: (1) The nitrogen atoms of the 2-aminopyrazine, 2-pyrazinylmethanol, and pyrazolopyrazine cores, as well as the carbonyl groups of the pyrimidinone and pyrazolopyrimidinone cores of allosteric inhibitors, act as acceptors to form hydrogen bonds with Arg111’s guanidine side chain. (2) The amino groups of the 2-aminopyrazine cores and the protonated nitrogen atoms of the pyrazolopyrazine cores of allosteric inhibitors act as donors to form hydrogen bonds with Glu250’s backbone carbonyl group. (3) The nitrogen atoms of the pyrimidinone and pyrazolopyrazine cores of allosteric inhibitors act as acceptors to form hydrogen bonds with Thr253’s hydroxyl side chain. (4) The primary amines of 4-methylpiperidin-4-amine from the polar regions of allosteric inhibitors act as donors to form hydrogen bonds with Phe113’s backbone carbonyl group. (5) Conjugated structures, such as o-dichlorobenzene from the hydrophobic regions of allosteric inhibitors, are able to form π-cation stacking interactions with Arg111’s guanidine side chain. (6) The fluorine atoms of 2-trifluoromethylpyridine from the hydrophobic regions of allosteric inhibitors are able to form halogen interactions with Gln257 and Gln495. (7) Conjugated structures, such as o-dichlorobenzene and 2-trifluoromethylpyridine from the hydrophobic regions of allosteric inhibitors, are able to form a hydrophobic interaction network with Thr219, Leu254, Gln257, Pro491, Lys492, and Gln495. Overall, the results of the high-frequency interaction fingerprint analysis (frequencies > 0.40) are in perfect agreement with the key residues (ΔG ≤ −1.26 kJ/mol) analyzed by MM/GBSA calculations.

In conclusion, through MM/GBSA calculations and high-frequency interaction analysis, we evaluated the thermodynamic properties of various inhibitors bound to SHP2’s allosteric site. Additionally, we identified key residues and interaction fingerprints that stabilize the complex and are involved in SHP2’s allosteric regulation. This provides a solid foundation for understanding the allosteric regulatory mechanisms of SHP2.

### 2.2. The Interaction Between the N-SH2 and PTP Domains

The interface interaction between the N-SH2 and PTP domains is closely related to the allosteric effect of SHP2. A weakening of the interaction between the N-SH2 and PTP domains can lead to their separation, exposing the catalytic active site (orthosteric site) of the PTP domain, thereby converting SHP2 from the inactive to the active state (Figure 1). To analyze the effects of allosteric inhibitors on the interface interaction between the N-SH2 and PTP domains, as well as on the catalytic active site, we calculated the binding free energy (ΔG_bind_) between the N-SH2 and PTP domains using the MM/GBSA method based on molecular dynamics simulation trajectories (50~100 ns) of 45 allosteric inhibitor-bound SHP2 and apo-SHP2.

**Figure 4:**
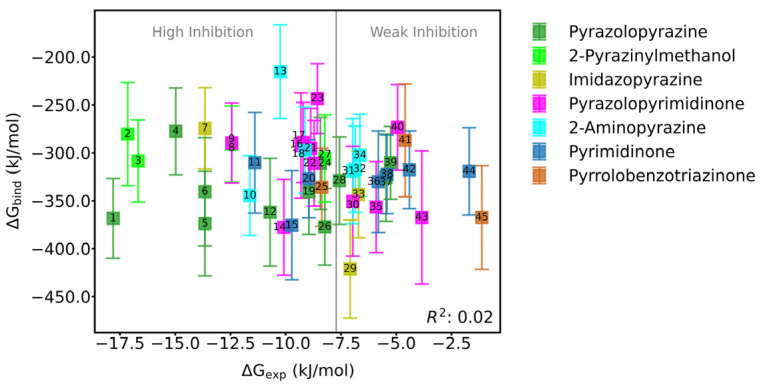
Scatter plot of binding free energy (ΔG_bind_) between N-SH2 and PTP domains vs. experimental inhibitory activity (ΔG_exp_) of allosteric inhibitors. The scatters are color-coded according to the mother nucleus of the allosteric inhibitors. The structural details are given in Figure S1.

As shown in Table S2 and Figure 4, the binding free energies between N-SH2 and PTP domains of allosteric inhibitor-bound SHP2 ranged from −421.25 to −215.39 kJ/mol. The binding free energies between the N-SH2 and PTP domains of apo-SHP2 ranged from −363.59 to −291.50 kJ/mol. This indicates that allosteric inhibitors with different structural characteristics can influence the interaction between the N-SH2 and PTP domains. Similarly, we attempted to establish a correlation between the binding free energy of the N-SH2/PTP domains and the experimental inhibitory activity (ΔG_exp_ = RT × ln(IC50)). However, the correlation coefficient (R^2^) was only 0.02, indicating no correlation between the interaction strength of the N-SH2 and PTP domains and the experimental inhibitory activity.

**Figure 5:**
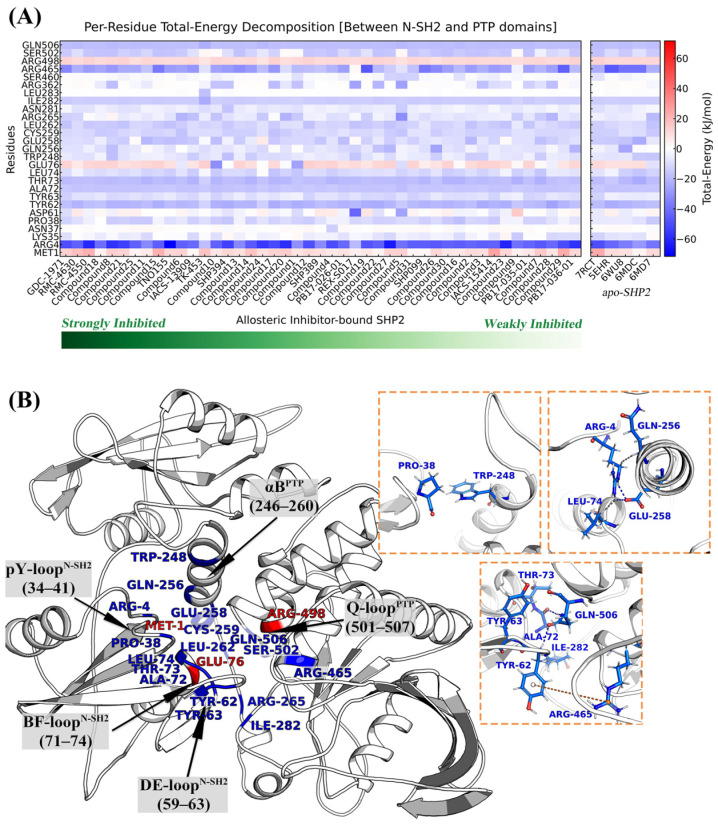
Interaction energies between N-SH2 and PTP domain residues (**A**) and the interaction analysis between N-SH2 and PTP domains (**B**).

By decomposing the binding free energy into the interaction energies between residue pairs of the N-SH2 and PTP domains (Figure 5A and Supplementary Figure S6), we identified the key residues and interactions that maintain the binding of the N-SH2 and PTP domains. In the allosteric inhibitor-bound SHP2, Arg4, Pro38, Tyr62, Tyr63, Ala72, Thr73, Leu74, Trp248, Glu258, Cys259, Leu262, Aag265, Ile282, Arg465, Ser502, and Gln506 make significantly positive contributions to the binding between the N-SH2 and PTP domains (≤−8.37 kJ/mol). These residues facilitate the maintenance of N-SH2/PTP interfacial interactions, thereby blocking the transition of SHP2 from the inactive to the active state, which is also associated with the allosteric regulatory.

Analysis of the distribution of these residues in the spatial structure of SHP2 (Figure 5B) reveals that the interface interactions between the N-SH2 and PTP domains primarily focused on the contacts between Py-loop^N-SH2^ and BF-loop^N-SH2^ with αB^PTP^, as well as between BF-loop^N-SH2^ and DEloop^N-SH2^ with Q-loop^PTP^. Among them, Q-loop^PTP^ represents the catalytic active site (orthosteric site) of SHP2, located distally from the inhibitor binding site (allosteric site), while αB^PTP^ is situated proximally to the inhibitor binding site. Notably, Arg4, Tyr62, Ala72, Arg465, and Gln506 make the most significant energy contributions to the N-SH2/PTP interface binding, with average values of −45.92, −20.09, −18.84, −23.86, and −18.46 kJ/mol, respectively. Interaction analysis (Figure 5B) reveals that these residues form strong interactions, such as hydrogen bonds and electrostatic interactions, to stabilize the N-SH2/PTP interface: (1) Arg4 forms a hydrogen bond with Glu258; (2) Gln506 forms hydrogen bonds with Tyr62 and Ala72; (3) Arg465 forms electrostatic or π-cation stacking interactions with Tyr62.

In conclusion, we calculated the thermodynamic data of N-SH2/PTP interfacial binding in the allosteric inhibitor-bound state by MM/GBSA method. Meanwhile, key residues and interactions at the N-SH2/PTP domain interface and catalytically active sites were identified, providing clues for further understanding of the allosteric regulatory mechanism of SHP2.

### 2.3. Machine Learning Based on Data from Equilibrium Molecular Dynamics Simulations

Our study encountered certain challenges. While equilibrium molecular dynamics simulations have provided insights into the thermodynamic steady-state structures of complexes, such as analyzing residues with high binding free energy contributions and interaction fingerprints with high frequencies of occurrence, they have sacrificed a substantial amount of dynamic data, thereby limiting the in-depth exploration of structural features related to allosteric effects. In addition, relying only on the thermodynamic data of allosteric inhibitor binding or N-SH2/PTP interfacial interactions to characterize experimental inhibitory activity has its limitations, as the kinetic processes of SHP2 conformational transitions are not taken into account. This makes it difficult to efficiently establish and generalize the “structure–activity relationship” of allosteric inhibitors. Beyond thermodynamic properties, the kinetic properties of SHP2 conformational transitions should also be fully considered. To address this, we turned to machine learning methods to analyze data from equilibrium molecular dynamics simulations. Unlike conventional analysis methods based on steady-state structural and thermodynamic information, machine learning offers unique advantages in revealing the characteristics of complex and dynamic structural changes.

In this analytical approach, we extracted a total of 45,000 frames of trajectory analysis data (with 86 features), ligand–receptor interaction fingerprints (with 107 features), and residue contact matrices (with 15,278 features) from equilibrium molecular dynamics simulations of 45 allosteric inhibitor-bound SHP2. For each allosteric inhibitor, 1000 frames were evenly extracted from the 0~100 ns simulation trajectory. These data cover all the dynamic structural information of the SHP2 complex in different variant inhibitor-bound states. Further, using the experimental activity data (IC50 values) of each allosteric inhibitor as the target variable, we constructed multiple machine learning classification and regression models such as XGBoost (extreme gradient boosting), KNN (K-nearest neighbors), LR (logistic regression), RF (random forests), SVM (support vector machines), and DT (decision trees). All models were developed based on the training dataset obtained by random splitting (70% of the 45,000 frames) and evaluated for their ability to predict IC50 values using the test dataset (the remaining 30%). For classification models, we evaluated the models based on accuracy, precision, recall, and F1 index. For the regression model, we evaluated the models based on the correlation coefficient (R^2^), mean square error (MSE), root mean square error (RMSE), and mean square absolute error (MAE). Tables S3 and S4 show all the machine learning models constructed based on equilibrium molecular dynamics simulation data and their evaluation results. Overall, the machine learning classification models (accuracy ranging from 0.61 to 0.84) outperformed the regression models (R^2^ ranging from 0.41 to 0.71) in predicting inhibitory activity (IC50). Among the classification models, the XGBoost exhibited the best performance among all three types of input features. Specifically, the accuracy of using trajectory analysis data, interaction fingerprints, and residue contact matrices as input features for the XGBoost classifier was 0.84, 0.84, and 0.82, respectively. These results indicate that all three feature types capture critical information for classification tasks, enabling better discrimination of inhibitory activity. Consequently, we selected the XGBoost classification model and used the SHAP method to enhance the model’s interpretability.

The SHAP method was used to analyze the impact of individual samples from each structural feature on experimental inhibitory activity. Specifically, by calculating the mean absolute SHAP values for each structural feature, we quantified its correlation with experimental inhibitory activity. The larger the mean absolute SHAP value of a structural feature, the more important that structural feature is for model prediction, indicating a stronger correlation with experimental inhibitory activity. Additionally, analyzing the distribution of SHAP values for all samples of a particular structural feature helps us understand how variations in that structural feature affects the experimental inhibitory activity. A positive SHAP value indicates that the structural feature enhances inhibitory activity (positive impact), whereas a negative SHAP value suggests it weakens inhibitory activity (negative impact).

#### 2.3.1. Trajectory Analysis Data as Input Conformational Features

**Figure 6:**
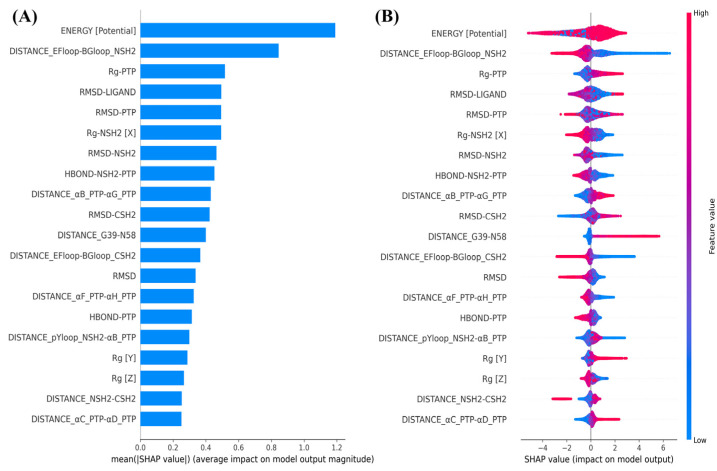
Top 20 most important simulated trajectory analysis data (**A**) and their SHAP value distribution (**B**).

To identify which dynamic structural features of SHP2 are influenced by allosteric inhibitors, we used various trajectory analysis data from molecular dynamics simulations (such as RMSD, Rg, SASA, backbone dihedral angles, hydrogen bond statistics, inter-domain distances, and distances between secondary structures) as input features. The experimental inhibitory activity (IC50 values) of each inhibitor against SHP2 served as the target variable, allowing us to build machine learning models. Figure 6 presents the top 20 trajectory analysis features, ranked by importance according to the mean absolute SHAP values, as well as the distribution of their SHAP values. These trajectory features show the strongest correlation with experimental inhibitory activity. The SHAP value distribution analysis reveals that an increase in features such as the radius of gyration of the PTP domain (Rg-PTP), the RMSD of the C-SH2 domain (RMSD-CSH2), the radius of gyration along the *Y*-axis (Rg [Y]), the interaction distance between αB and αG helices in the PTP domain (DISTANCE_αB_PTP-αG_PTP), the interaction distance between Gly39 and Asn58 (DISTANCE_G39-N58), and the interaction distance between αC and αD helices in the PTP domain (DISTANCE_αC_PTP-αD_PTP) (indicated by red sample points) enhance the experimental inhibitory activity (positive SHAP values). Conversely, a reduction in features such as the interaction distance between the EF-loop and BG-loop in the N-SH2 domain (DISTANCE_EFloop-BGloop_NSH2), the interaction distance between the EF-loop and BG-loop in the C-SH2 domain (DISTANCE_EFloop-BGloop_CSH2), and the RMSD of the N-SH2 domain (RMSD-NSH2) (indicated by blue sample points) also leads to enhanced experimental inhibitory activity (positive SHAP values).

#### 2.3.2. Ligand–Receptor Interaction Fingerprints as Input Conformational Features

**Figure 7:**
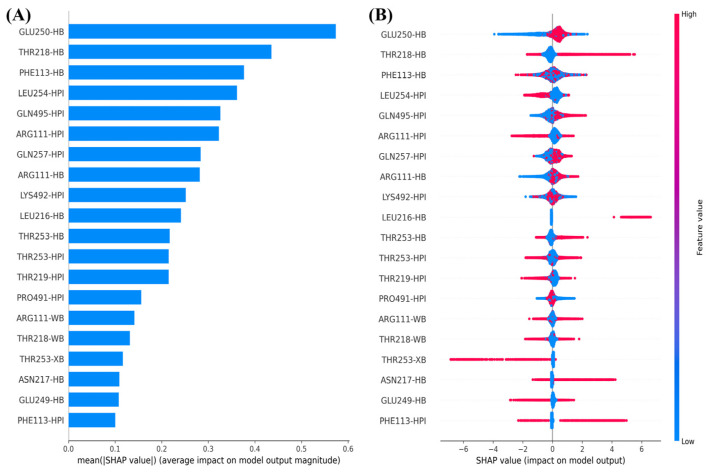
Top 20 most important ligand–receptor interaction fingerprints (**A**) and their SHAP value distribution (**B**).

To identify which interactions between allosteric inhibitors and SHP2 most effectively enhance experimental inhibitory activity, we used ligand–receptor interaction fingerprints as input features and the experimental inhibitory activity (IC50 values) of each inhibitor on SHP2 as the target variable to construct machine learning models. Figure 7 shows the top 20 ligand–receptor interaction fingerprint features, ranked by importance, and the distribution of their SHAP values. The SHAP value distribution suggests that hydrogen bonds (HB) of allosteric inhibitors with Glu250, Thr218, Arg111, and Leu216, as well as hydrophobic interactions (HPI) with Gln495 and Gln257 (indicated by red sample points), enhance the experimental inhibitory activity (positive SHAP values). Conversely, the absence of these interactions (indicated by blue sample points) leads to a reduction in experimental inhibitory activity (negative SHAP values).

#### 2.3.3. Residue Contact Matrix as Input Conformational Features

**Figure 8:**
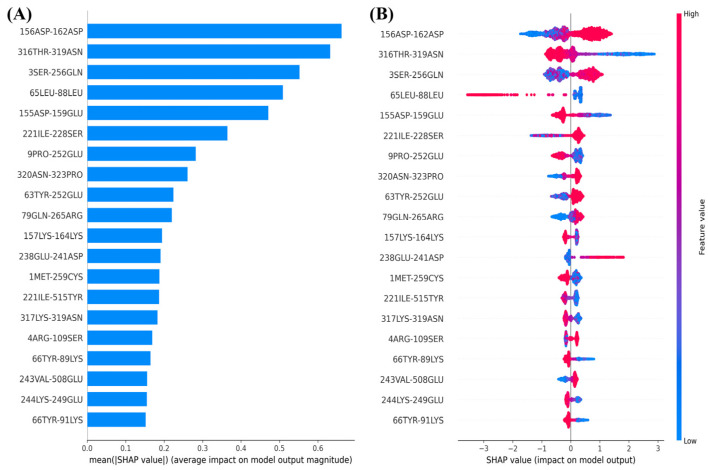
Top 20 most important contact residue pairs (**A**) and their SHAP value distribution (**B**).

To comprehensively analyze the relationship between the dynamic structural features of SHP2 and experimental inhibitory activity, we used the residue contact matrix (the shortest interaction distances between all pairs of residues) as input features and the experimental inhibitory activity (IC50 values) of each inhibitor on SHP2 as the target variable to construct machine learning models. Figure 8 presents the top 20 contact residue pairs ranked by importance, and their SHAP value distributions. The features “316THR-319ASN”, “65LEU-88LEU”, “155ASP-159GLU”, “9PRO-252GLU”, “1MET-259CYS”, “221ILE-515TYR”, and “317LYS-319ASN” represent the contact (interaction) distances between these residue pairs. The SHAP value distributions indicate that the strengthening of interactions between these residue pairs (indicated by blue sample points) leads to an increase in experimental inhibitory activity (positive SHAP values). Conversely, the weakening of interactions between residue pairs such as “156ASP-162ASP”, “3SER-256GLN”, “221ILE-228SER”, “320ASN-323PRO”, “63TYR-252GLU”, “79GLN-265ARG”, and “238GLU-241ASP” (indicated by red sample points) also enhance the experimental inhibitory activity (positive SHAP values).

In conclusion, by applying machine learning methods to the analysis of equilibrium molecular dynamics simulation data, we demonstrated the unique advantages of machine learning in revealing complex dynamic structural features. Our work not only addresses the limitations of traditional thermodynamic and static structural analyses but also enhances model interpretability through the SHAP method, enabling a quantitative and intuitive understanding of the relationship between key structural features and experimental inhibitory activity. By identifying and analyzing important trajectory data, ligand–receptor interaction fingerprints, and residue contact matrices, we have not only deepened our understanding of the allosteric regulation mechanisms of SHP2 but also provided new perspectives and methodologies for the future design of allosteric inhibitors.

### 2.4. Kinetic Model of Enzyme-Catalyzed Reaction of Allosteric Inhibitor-Bound SHP2

**Figure 9:**
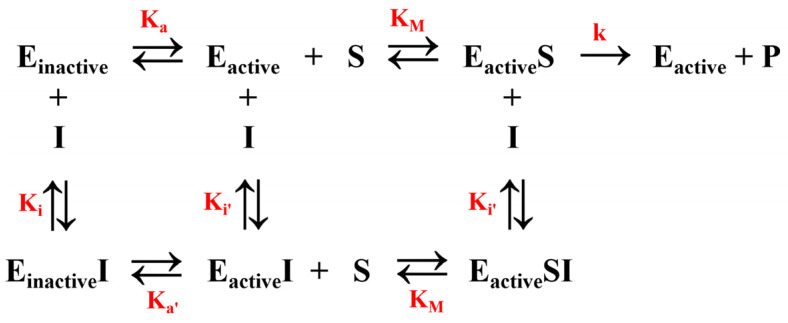
Kinetic model of enzyme-catalyzed reaction of allosteric inhibitor-bound SHP2.

In Section 2.1, we mentioned that it was difficult to establish a correlation between the predicted binding free energy (binding affinity) and the experimental inhibitory activity of the allosteric inhibitors, which may be related to the unique enzyme inhibition kinetics of SHP2. The chemical kinetic models of allosteric inhibitors may help to understand the mechanism of this series of SHP2 inhibitors. Figure 9 shows a kinetic model of enzyme-catalyzed reaction of allosteric inhibitor-bound SHP2, which is based on a chemical equilibrium between the active and inactive conformations. This equilibrium can be affected by allosteric inhibitor binding. Only the active conformational enzyme has catalytic activity, and the reaction follows a Michaelis–Menten kinetics [40].

The enzyme-catalyzed reaction rate V versus substrate concentration [S] was obtained by derivation through the Michaelis–Menten equation (the detailed derivation is given in Figure S7):

(1) $$\frac{1}{V}=\frac{K_{M}}{V_{\max}}(1+\frac{\left[I\right]}{K_{i^{\prime }}}+\frac{1}{K_{a}}+\frac{\left[I\right]}{K_{i^{\prime }}\;K_{a^{\prime }}})\frac{1}{\left[S\right]}+\frac{1}{V_{\max}}(1+\frac{\left[I\right]}{K_{i^{\prime }}})$$

Equation (1) is briefly written as follows:

(2) $$\frac{1}{V}=\frac{K_{M}}{V_{\max}}\frac{\alpha }{\left[S\right]}+\frac{\alpha \prime }{V_{\max}}$$

$$\mathrm{Here},\alpha =1+\frac{\left[I\right]}{K_{i^{\prime }}}+\frac{1}{K_{a}}+\frac{\left[I\right]}{K_{i^{\prime }}\;K_{a^{\prime }}}\begin{array}{cc} , & \alpha \prime =1+\frac{\left[I\right]}{K_{i^{\prime }}} \end{array}$$

where [I] is the concentration of allosteric inhibitors, [S] is the concentration of substrate, V_max_ is the maximal enzyme velocity, V is the fractional velocity in the presence of varying concentration of inhibitor, K_M_ is the Michaelis–Menten constant, K_i_ and K_i′_ are the dissociation constant of inhibitor, K_a_ and K_a′_ are equilibrium constants between the active and inactive states, and α and α′ are the interaction constants as a symbol of the affinity difference between inhibitor and the free enzyme or enzyme–substrate complex.

When an allosteric inhibitor is introduced into the system, the enzyme-catalyzed reaction equilibrium changes, and we use K_M,app_ and V_max,app_ to represent the observed kinetic parameters under the influence of the inhibitor:

(3) $$K_{M,\mathrm{app}}=\frac{{\alpha K}_{M}}{\alpha \prime }$$

(4) $$V_{\max,\mathrm{app}}=\frac{V_{\max}}{\alpha \prime }$$

From Equation (2), both α and α′ are greater than 1, and α is greater than α′. Thereby, V_max,app_ decreases curvilinearly with increasing concentration of the allosteric inhibitor, while K_M,app_ increases inversely. The allosteric inhibitor binding to SHP2 can exhibit noncompetitive (mixed) kinetic characteristics on the enzyme-catalyzed reaction, which is consistent with the results of the steady-state kinetic assays in the publication by Kai Tang et al [41]. It is known that a noncompetitive (mixed) inhibitor displays varied binding affinities toward both the free enzyme and the enzyme–substrate complex. The interaction constant α is commonly used to identify the binding preference of noncompetitive inhibitor. The trend of K_M,app_ in Equation (3) and V_max,app_ in Equation (4) suggests the favorable affinity of the allosteric inhibitors toward the free SHP2, rather than toward the SHP2–substrate complex, characterized by the interaction constant α > 1.

In addition, the interaction constant α of SHP2 exhibits two more subterms, 1/K_a_ and [I]/K_i′_K_a′_, in Equation (2) compared to the enzyme without the allosteric characteristics. This indicates that the observed kinetic parameter K_M,app_ is affected by the equilibrium constants K_a_ and K_a′_ between the active and inactive states of SHP2. Therefore, the key to understanding the mechanism of these inhibitors is to understand the principle of the active/inactive conformational equilibrium. To this end, in the next section, we chose to carry out enhanced sampling simulation of the SHP2 allosteric process and compare the free energy landscape of the inhibitor-bound and inhibitor-unbound states obtained by the enhanced sampling based on molecular dynamics simulation, to explain how inhibitors affect target allosteric processes.

### 2.5. Free Energy Landscapes and Kinetic Data for Allosteric Regulation Processes of SHP2

In the previous 100 ns conventional molecular dynamics simulations, we observed no significant change in SHP2 conformation, indicating the need for long-scale simulations or enhanced sampling. To gain insight into the activation mechanism of SHP2, we performed well-tempered meta-dynamics simulations (Meta-MD) to sample the conformational space of the enzyme. Considering the high computational cost of the Meta-MD method applied in this study, we selected only five representative inhibitor-bound (RMC-4550, IACS-13909, SHP389, SHP099, and Compound **7**) SHP2, as well as one apo-SHP2, and performed six sets of Meta-MD simulations. Among them, the five inhibitors represented a range of distinct structural scaffolds, each featuring different hydrophobic and polar regions, and their IC50 values spanned a wide range (from 0.00155 to 0.181 μM). Therefore, the selection of these inhibitors for meta-dynamics simulations enabled us to comprehensively explore how the inhibitors’ various structural features and potencies influenced SHP2 conformational transitions. For all six sets of meta-dynamics simulations, the initial structures were the inactive conformation of SHP2. We used the RMSD values of the entire protein heavy atom of the sampled structure relative to the crystal structure of SHP2’s active conformation (PDB ID: 6CRF) and the distance between the D’E loop of the N-SH2 domain (heavy atoms of residues 58–72) and the catalytic site of the PTP domain (heavy atoms of residues 276–288, 361–366, 421–431, 458–465, 501–507) as the collective variables (CVs) for each set of simulations. After 400 ns Meta-MD simulation, the collective variables were used as reaction coordinates to construct two-dimensional free energy landscapes of SHP2 conformational transitions. To ensure the convergence of the simulation results, we additionally generated free energy landscapes from Meta-MD simulation trajectories of the same system at 300 ns, 350 ns, and 400 ns (Figure S8). The absence of significant differences among these free energy landscapes demonstrated that the 400 ns Meta-MD simulation was sufficient to achieve satisfactory convergence.

**Figure 10:**
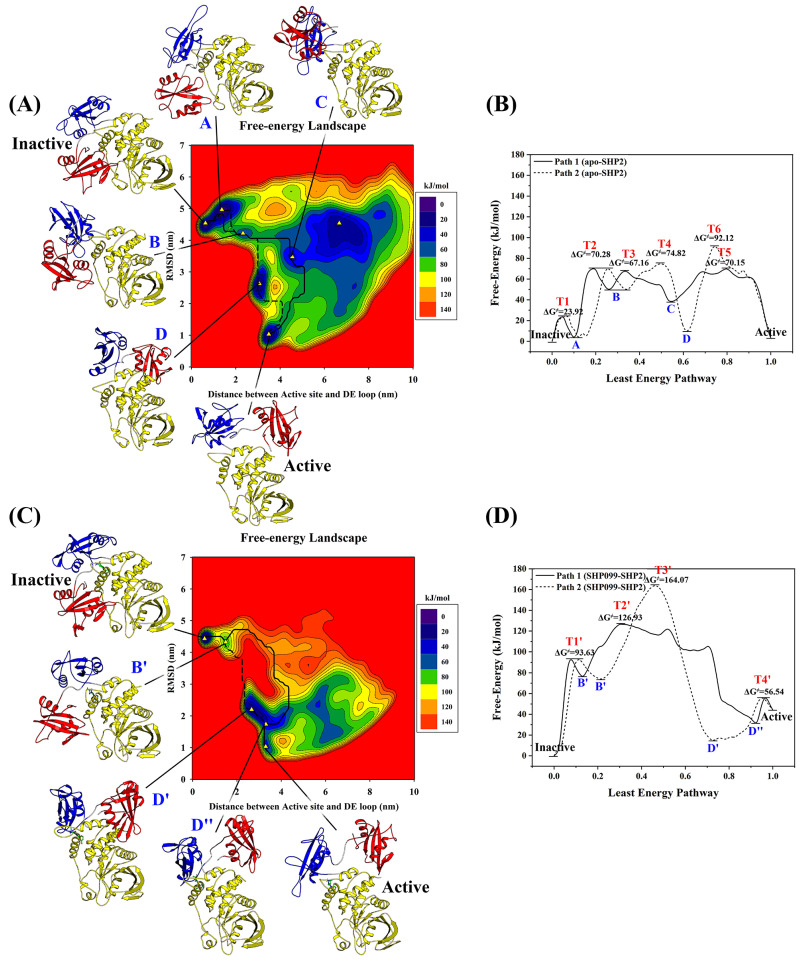
Free energy landscapes of apo-SHP2 and SHP099-bound SHP2 activation processes (**A**,**C**) and their minimum free energy paths (**B**,**D**).

On free energy landscapes of apo-SHP2 constructed along collective variables (Figure 10A), Meta-MD simulations captured two stable states (active and inactive), five substable states (A, B, C, D, and E) corresponding to low free energy wells, and six transition states (T1, T2, T3, T4, T5, and T6) corresponding to free energy barriers. Among them, the inactive state of apo-SHP2 is at the global energy minimum (defined as the zero point) of the free energy landscape, which is consistent with the crystallographic structure resolution results, and indicates that the inactive state of SHP2 protein is thermodynamically most stable on the free energy landscape. On the free energy landscape of inhibitor-bound SHP2 (Figure 10C and Figure S9), the conformational space of SHP2 is significantly reduced. Taking SHP099 as an example, apart from the active and inactive states, Meta-MD simulations captured only two substates (B′ and D′) corresponding to low free energy wells and four transition states (T1′, T2′, T3′, and T4′) corresponding to free energy barriers. The inactive state of the inhibitor-bound SHP2 also remained at the global energy minimum of the free energy landscape (defined as the zero point), re-emphasizing the thermodynamic stability of the inactive state of SHP2 protein. However, the free energy of the active state of the inhibitor-bound SHP2 increased by 40.87 kJ/mol compared to apo-SHP2, demonstrating that the inhibitor substantially attenuated the thermodynamic stability of the active state of SHP2.

Further, to determine kinetic data for the enzyme activation pathways of apo-SHP2 and SHP099-bound SHP2, the MULE software 0.2 and the Dijkstra algorithm were used to scan for the minimum free energy pathways linking the active and inactive states. On the free energy landscape of apo-SHP2 (Figure 10A), two pathways (Path I and Path II) of SHP2 transitions from inactive to active conformations were obtained, corresponding to the directions along “Inactive–(T1)–A–(T2)–B–(T3)–C–(T5)–Active” and “Inactive–(T1)–A–(T2)–B–(T4)–D–(T6)–Active”, respectively. Their one-dimensional free-energy profiles are shown in Figure 10B, and the highest energy barriers that must be overcome for SHP2 to transform into the active state are 70.28 and 92.12 kJ/mol (T2 and T6), respectively. On the free energy landscape of SHP099-bound SHP2 (Figure 10C), two pathways (Path I and Path II) similar to that of apo-SHP2 were captured, corresponding to the directions along “Inactive–(T1′)–B′–(T2′)–D″–(T4′)–Active” and “Inactive–(T1′)–B′–(T3′)–D′–(T4′)–Active”, respectively. Their one-dimensional free-energy profiles are shown in Figure 10D, and the highest energy barriers that must be overcome for SHP2 to transform into the active state are 126.93 and 164.07 kJ/mol (T2′ and T3′), respectively. Obviously, the binding of SHP099 resulted in a rise of 56.65 and 71.95 kJ/mol in the highest energy barriers of the two pathways, and SHP099-bound SHP2 had to overcome a higher free energy barrier during enzyme activation.

Using the same methodology, we analyzed the lowest free energy barriers of the SHP2 from the inactive to the active state in the bound states of four other representative inhibitors (RMC-4550, IACS-13909, SHP389, and Compound **7**) (Figure S9). The minimum free energy barriers were found to be 131.90, 126.81, 99.19, and 120.76 kJ/mol, respectively, which are significantly higher than the minimum free energy barrier of the apo-SHP2, measured at 70.28 kJ/mol. This suggests a significant inhibitory effect of the allosteric inhibitor on the kinetic process of SHP2 activation. Furthermore, based on transition state theory, we calculated the activation rate k_active_ and inactivation rate k_inactive_ of SHP2, as well as the equilibrium constant K_a′_ between the active and inactive states. The results, presented in Table 1, indicate that the kinetic data of the inhibitor-bound SHP2 during the allosteric process has changed significantly compared to the apo-SHP2. Specifically, the inhibitors significantly increase the allosteric activation free energy barrier Δ$G_{active}^{\neq }$ which in turn drastically reduces the allosteric rate constant k_active_, thereby greatly inhibiting the activation of SHP2. We attempted to establish a correlation between the calculated value 1/K_i′_K_a′_ and the experimental inhibitory activity. The correlation coefficient (R^2^) was 0.75, which suggests that the thermodynamic properties of inhibitor binding, K_i′_, are jointly determined with the kinetic properties of SHP2’s conformational change, K_a′_.

In summary, through Meta-MD simulations, we systematically revealed the transition process of SHP2 from the inactive to the active state and provided a detailed analysis of the free energy landscape during this process. The Meta-MD simulations not only analyzed various stable, metastable, and transition state conformations of both apo-SHP2 and inhibitor-bound SHP2 but also predicted the kinetic data of the allosteric process of SHP2. These findings not only provide important theoretical insights for understanding the allosteric regulation of SHP2 but also offer new ideas and directions for the development of allosteric inhibitors targeting SHP2.

### 2.6. Machine Learning Based on Meta-Dynamics Simulation Data

Meta-dynamics simulations have helped us expand the conformational space of SHP2 and successfully elucidated the kinetic regulatory mechanism of inhibitors on SHP2. However, the large-scale and high-dimensional structural information in the Meta-MD simulation trajectories poses challenges for effectively identifying key structural features that regulate SHP2 conformational transitions. Therefore, we applied a comprehensive approach of interpretable machine learning to further analyze the Meta-MD simulations trajectories. We used 40,000 frames of the residue contact matrix data (the shortest interaction distances between all pairs of residues) from the meta-dynamics simulations trajectories as input features and the free energy data as the objective function to construct an XGBoost machine learning regression model (Table S5). Additionally, we employed the SHAP method to interpret and identify the important structural features within the Meta-MD simulations trajectories.

**Figure 11:**
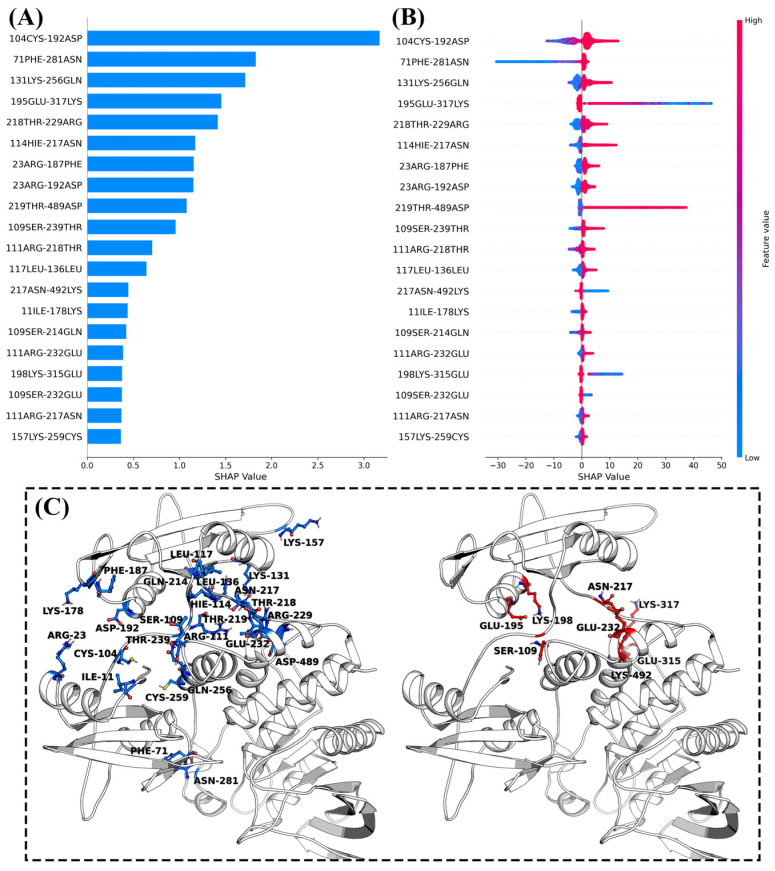
(**A**) Top 20 most important contact residue pairs identified from the machine learning model trained on the meta-dynamics simulations of apo-SHP2; (**B**) distribution of SHAP values of top 20 most important contact residue pairs; (**C**) spatial distribution of top 20 most important contact residue pairs in the three-dimensional structure of SHP2.

Figure 11B illustrates the distribution intervals of SHAP values for the top 20 most important features ranked in the model trained on the Meta-MD trajectories of apo-SHP2. For interactions between residue pairs “104CYS-192ASP”, “71PHE-281ASN”, “131LYS-256GLN”, “218THR-229ARG”, “114HIE-217ASN”, “23ARG-187PHE”, “23ARG-192ASP”, “219THR-489ASP”, “109SER-239THR”, “111ARG-218THR”, “117LEU-136LEU”, “11ILE-178LYS”, “109SER-214GLN”, “111ARG-232GLU”, “111ARG-217ASN”, and “157LYS-259CYS”, their SHAP value distributions show that the enhancement of these interactions (depicted by blue points) contribute to SHP2’s tendency to maintain a low-energy conformation (SHAP values below zero). Conversely, the attenuation of these interactions (depicted by red points) lead to a rapid transition of SHP2 to a high-energy conformation (SHAP values above zero). Thus, apo-SHP2 stabilizes the inactive conformation mainly through interactions between residue pairs such as “104CYS-192ASP”.

**Figure 12:**
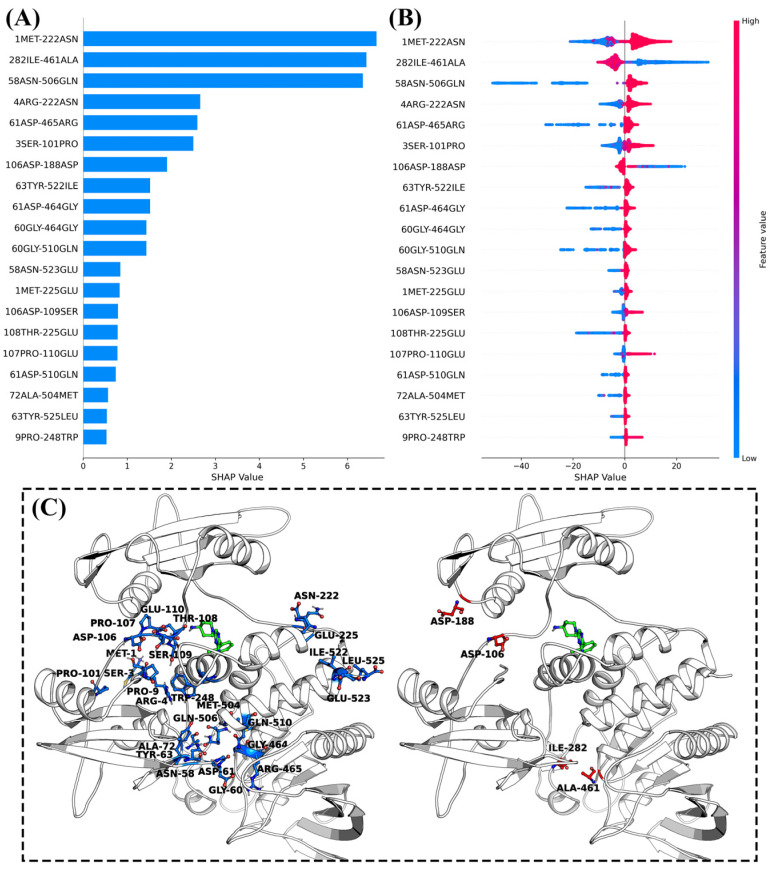
(**A**) Top 20 most important contact residue pairs identified from the machine learning model trained on meta-dynamics simulations of SHP099-bound SHP2; (**B**) distribution of SHAP values of top 20 most important contact residue pairs; (**C**) spatial distribution of the top 20 most important contact residue pairs in the three-dimensional structure of SHP2.

Figure 12B displays the distribution range of SHAP values for the top 20 most important features in the model trained on the Meta-MD trajectories of inhibitor-bound SHP2. For interactions between residue pairs “1MET-222ASN”, “58ASN-506GLN”, “4ARG-222ASN”, “61ASP-465ARG”, “3SER-101PRO”, “63TYR-522ILE”, “61ASP-464GLY”, “60GLY-464GLY”, “60GLY-510GLN”, “58ASN-523GLU”, “1MET-225GLU”, “106ASP-109SER”, “108THR-225GLU”, “107PRO-110GLU”, “61ASP-510GLN”, “72ALA-504MET”, “63TYR-525LEU”, and “9PRO-248TRP”, their SHAP value distributions show that the enhancement of these interactions, which are indicated by the blue points, allow SHP2 to be in a low-energy conformation (SHAP values below zero). Conversely, the weakening of these interactions (indicated by red points) leads to a rapid transition of SHP2 to a high free energy conformation (positive SHAP value). It can be deduced that the inhibitor increases the stability of the inactive conformation of SHP2 mainly by enhancing the interactions between residue pairs such as “1MET-222ASN”, thus inhibiting the allosteric (activation) process of SHP2.

## 3. Materials and Methods

### 3.1. Allosteric Inhibitor Dataset

In this study, we created an SHP2 allosteric inhibitor dataset, containing a total of 45 compounds with IC50 values ranging from 0.001 to 0.645 μM. Among them, compounds with IC50 values higher than 0.050 μM were defined as weakly active inhibitors, while compounds with IC50 values lower than 0.050 μM were defined as strongly active inhibitors. Some of the allosteric inhibitors have a co-crystal structure with SHP2 in the RCSB Protein Data Bank, which can be directly used as the initial structure for running molecular dynamics simulations. The structures of rest of allosteric inhibitors in complex with SHP2 were predicted by molecular docking and were further used as initial structures for running molecular dynamics simulations. Detailed information on the 2D structures, IC50 values, co-crystal protein ID numbers, and docking scores of all allosteric inhibitors are shown in Figure S1.

**Figure 13:**
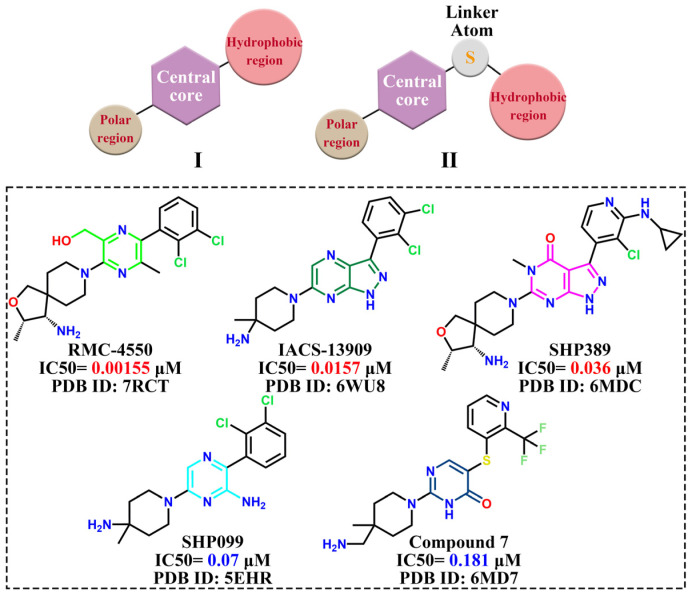
Structural generalizations of allosteric inhibitors and five representative inhibitors.

From the perspective of 2D structure, these allosteric inhibitors have two structural generalizations (Figure 13): Type I, which consists of the hydrophobic region, the mother nucleus, and the polar region; and Type II, which adds connecting atoms (sulfur atoms) between the hydrophobic region and the structural mother nucleus. Specifically, the mother nucleus includes structures such as the 2-aminopyrazine, pyrimidinone, 2-pyrazinylmethanol, pyrazolopyrimidinone, pyrrolobenzotriazinone, pyrazolopyrazine, and imidazolopyrazine. The hydrophobic region includes structures such as chlorobenzene, o-dichlorobenzene, 2-trifluoromethylpyridine, and 2-amino-3-chloropyridine. The polar region includes structures such as 4-methylpiperidin-4-amine and five-membered spirocycles with primary amines (3-methyl-2-oxa-8-azaspiro[4.5]decan-4-amine and spiro[3H-1-benzofuran-2,4′-piperidine]-3-amine).

### 3.2. Molecular Docking

In this study, since some allosteric inhibitors with SHP2 proteins lacked co-crystal structure, we used AutoDock Vina software 1.2.0 for molecular docking to predict the best binding conformations of these allosteric inhibitors with SHP2 [42]. Firstly, the structure files of allosteric inhibitors and SHP2 protein were all subjected to the addition of Gasteiger–Hückel charges, the merging of nonpolar hydrogen atoms, and the setting up of rotatable bonds by AutoDockTools software. The σ-bonds between heavy atoms in each allosteric inhibitor structure were all set as rotatable bonds, and SHP2 protein was considered as a rigid structure. After that, a 50 × 50 × 50 Å docking box was set at the junction of the C-SH2 and PTP domains of SHP2 by the AutoDock Vina program. Conformational search and energy optimization in docking box were performed for the allosteric inhibitor, and the calculation was terminated after obtaining the best binding conformation of the allosteric inhibitor to SHP2 (the conformation with the highest docking score). Other parameters used to run the AutoDock Vina program during the calculation are default values. Finally, the best binding conformation of the allosteric inhibitor to SHP2 was used as the initial structure for performing molecular dynamics simulations.

### 3.3. Equilibrium Molecular Dynamics Simulations

Equilibrium molecular dynamics simulations of apo-SHP2 and allosteric inhibitor-bound SHP2 in the inactive state were performed. Before the simulation run, the H++ online server was used to compute the pK value of ionizable groups in SHP2 protein and add the missing hydrogen atoms of SHP2 according to the ambient pH [43]. In addition, structural optimization of each allosteric inhibitor was conducted with B3LYP/6-31G* using Gaussian09 software package D.01 [44]. The atomic partial charges for each allosteric inhibitor was the restrained electrostatic potential (RESP) charges determined by fitting with Antechamber and Acpype program [45]. The FF99SB and GAFF force fields were further selected to describe the SHP2 protein residues and allosteric inhibitor structures, respectively [46,47]. The apo-SHP2 and allosteric inhibitor-bound SHP2 were immersed in an octahedral box of TIP3P waters, setting a 12 Å minimal distance from the protein’s surface to the box [48]. The Na^+^ and Cl^−^ ions were also added to the solvent to keep the system neutral. During the simulations, the short-range electrostatic cutoff was 1.0 nm and the short-range van der Waals cutoff was 1.0 nm. The long-range electrostatic interactions were handled using the particle mesh ewald (PME) method [49] and all bonds involving hydrogen atoms were constrained using the SHAKE algorithm with a 2 fs time step [50].

GROMACS software version 2019.6 was used to accelerate all the MD simulations [51,52]. First, each SHP2 complex system was minimized with 20,000 steps of the steepest descent method. Subsequently, the system was then heated to 310 K during a 400 ps NVT (constant number of particles, volume, and temperature) simulation with a 2 fs time step. The pressure was then equilibrated to 1 atm during a 400 ps NPT (constant number of particles, pressure, and temperature) simulation with a 2 fs time step. The time constant for the temperature and pressure coupling was kept at 0.1 and 2.0 ps, respectively. Both temperature and pressure were regulated by V-rescale, a modified Berendsen thermostat, and the pressure was regulated by the Parrinello–Rahman method. In addition, the first 200 ps of the heating and pressure equilibrium simulations were positionally constrained for all heavy atoms of SHP2 with a force constant of 1000 kJ/mol/nm^2^. Finally, the 100 ns MD simulation was officially carried out.

### 3.4. Meta-Dynamics Simulations

In order to thoroughly investigate the SHP2 conformational transition mechanism, we explored the conformational change process of apo-SHP2 and five allosteric inhibitor-bound (RMC-4550, IACS-13909, SHP389, SHP099, and Compound **7**) SHP2 from the inactive to the active state by using meta-dynamics (Meta-MD) simulations [39] and plotted the free energy landscapes of the process. To ensure that SHP2 conformation is not trapped in the local minima during the simulations, we carried out meta-dynamics simulations using GROMACS software version 2019.6 and PLUMED 2 [53]. The initial structures for the meta-dynamics simulations were obtained from the last frame of the equilibrium molecular dynamics simulations. All meta-dynamics simulations were carried out in the NVT ensemble for 400 ns. The well-tempered meta-dynamics scheme was used to ensure a smooth convergence of the free energy landscape [39]. The collective variables (CVs) were chosen based on the experimental observations, including the RMSD values of the entire protein heavy atom of the sampled structure relative to the crystal structure of SHP2’s active conformation (PDB ID: 6CRF) and the distance between the D’E loop of the N-SH2 domain (heavy atoms of residues 58–72) and the catalytic site of the PTP domain (heavy atoms of residues 276–288, 361–366, 421–431, 458–465, 501–507). For the RMSD value and distance CVs, the Gaussian width was set to 0.30 nm, and the boundaries were set from 0 to 10 nm to prevent the protein from being pushed to biologically irrelevant conformations. The starting height of the Gaussian potential was set to 1.20 kJ/mol, and the Gaussians were deposited every 1 ps.

### 3.5. Conventional Analytics

After equilibration molecular dynamics, we carried out trajectory analysis by the GROMACS software package, including the calculation of root mean square deviation (RMSD) of the whole trajectory, radius of gyration (Rg), solvent-accessibility surface area (SASA), dihedral angle of the protein backbone Cα, hydrogen bond statistics, inter-domain distances, and inter-secondary structure distances. In addition, we calculated the binding free energy between the allosteric inhibitor and SHP2, as well as the interaction energies between the inhibitor and each residue of SHP2, using the Molecular Mechanics/Generalized Born Surface Area (MM/GBSA) method [32,33,38]. The MM/GBSA method is based on the following equations for calculating the binding free energy:

(5) $$\begin{array}{ll} {\Delta G}_{\mathrm{bind}} & {=G}_{\mathrm{complex}}{\;-\;(G}_{\mathrm{protein}}{+G}_{\mathrm{ligand}})\\ & {=\Delta E}_{\mathrm{MM}}{+\Delta G}_{\mathrm{solvation}}-T\Delta S\\ & {=\Delta E}_{\mathrm{MM}}{+\Delta G}_{\mathrm{GB}}{+\Delta G}_{\mathrm{SA}}\;-T\Delta S\\ & {=\Delta E}_{\mathrm{vdw}}{+\Delta E}_{\mathrm{ele}}{+\Delta G}_{\mathrm{GB}}{+\Delta G}_{\mathrm{SA}}\;-T\Delta S\\ & \approx {\;\Delta E}_{\mathrm{vdw}}{+\Delta E}_{\mathrm{ele}}{+\Delta G}_{\mathrm{GB}}{+\Delta G}_{\mathrm{SA}} \end{array}$$

in which ΔG_bind_ represents the binding free energy in the solution consisting of the molecular mechanic’s free energy (ΔE_MM_), the conformational entropic effect to binding (−TΔS) in the gas phase, and the solvation free energy containing polar contribution (ΔG_GB_) and nonpolar contribution (ΔG_SA_). Among them, the ΔE_MM_ term included electrostatic (ΔE_ele_) and van der Waals (ΔE_vdw_) energies, which were modeled using the Coulomb and Lennard-Jones (LJ) potential functions, respectively. The polar contribution to the solvation free energy (ΔG_GB_) was derived by fitting through the Generalized Born (GB) model, with the solvent and solute dielectric constants set to 80 and 4, respectively [54]. The nonpolar contribution to the solvation free energy (ΔG_SA_) was obtained by linear fitting of the equation ΔG_SA_ = γ × ΔSASA, where γ is the surface tension with a value of 0.02267 kJ mol^−1^ Å^−2^ [55,56]. The conformational entropy change (ΔS) was typically estimated by a normal mode analysis with the nmode module [57]. However, the entropy estimation based on computationally expensive normal mode analysis tended to have a large margin of error, which introduced significant uncertainty into the prediction results of the MMGBSA method [57,58,59]. Additionally, the 45 allosteric inhibitors in this study exhibited similar structural characteristics and binding poses to SHP2, and their conformational entropy could be ignored for predicting the relative binding free energies. Therefore, we omitted the contribution of conformational entropy (−TΔS) to the binding free energy in this study.

### 3.6. Machine Learning Analytics

Although molecular dynamics simulations allow us to obtain extensive sampling of various conformations of SHP2, the high dimensionality of the conformational space prevents the effective identification of key structural factors that regulate the conformational changes in SHP2. To address this, we extracted trajectory analysis data, ligand–receptor interaction fingerprints, and residue contact matrices from each frame of the simulation trajectories as input conformational features, and employed various models, such as XGBoost (extreme gradient boosting) [25], KNN (K-nearest neighbors) [60], LR (logistic regression) [61], RF (random forests) [62], SVM (support vector machines) [63], and DT (decision trees) [64], to learn the correlation between the conformational features and the labels of the free energy data or inhibitory activity data (IC50). We carried out four label annotation and learning schemes:

(1) Trajectory analysis data (RMSD, Rg, SASA, protein backbone dihedral angles, hydrogen bond statistics, inter-domain distances, and inter-secondary structure distances) from equilibrium molecular dynamics simulations of 45 inhibitor-bound SHP2 were used as input features, and the inhibitory activity data (IC50 values) of each inhibitor were used as annotation labels to construct various types of classification and regression models in machine learning.
(2) Ligand–receptor interaction fingerprints were extracted from the equilibrium molecular dynamics simulation trajectories of 45 inhibitor-bound SHP2 as input features, and the activity data (IC50 values) of each inhibitor were used as annotation labels to construct various types of classification and regression models in machine learning.
(3) Residue contact matrices were extracted from the equilibrium molecular dynamics simulation trajectories of 45 inhibitor-bound SHP2 as input features, and the activity data (IC50 values) of each inhibitor were used as annotation labels to construct various types of classification and regression models in machine learning.
(4) Residue contact matrices were extracted from meta-dynamics simulation trajectories of SHP2 conformational transitions as input features, and free energy landscape (free energy) data were used as annotation labels to construct various types of regression models in machine learning.

For Schemes 1~3, a total of 45,000 frames of conformational features (trajectory analysis data, ligand–receptor interaction fingerprints, and residue contact matrices) were extracted from equilibrium molecular dynamics simulation trajectories of 45 allosteric inhibitor-bound SHP2, with 1000 frames per inhibitor. For Scheme 4, 40,000 frames of residue contact matrices were extracted from the meta-dynamics simulation trajectories of SHP2 conformational transitions. The residue contact matrix of each frame was the minimum distance between heavy atoms of residue pairs within a range of 6.5 Å. The interaction fingerprints of each frame were grouped into seven categories of protein–ligand contacts: hydrophobic interactions (HPI), hydrogen bond (HB), halogen bond (XB), π-stacking (PST), π-cation (PIC), salt bridges (SB), and water bridges interactions (WB). Each category was assigned a value of 1 or 0, depending on whether the contact type was present or not. To prevent overfitting, 70% of these frames were allocated for the training dataset, while the remaining 30% were reserved for the test dataset. The model was built using the training dataset and evaluated on the test dataset by accuracy, precision, recall, F1 metrics, correlation coefficient (R^2^), mean square error (MSE), root mean square error (RMSE), and mean square absolute error (MAE) to understand its actual performance on independent data. The results show that the XGboost model performed best on each schemes, as we mainly applied and discuss the XGboost model. The XGBoost model was implemented using the scikit-learn package 1.3.0, and its hyperparameters were selected via a grid search strategy. After optimization, we chose a “learning rate” of 0.1, “n_estimators” of 10,000, “max_depth” of 11, and “min_child_weight” of 4 as a balance between overfitting and underfitting. To assess the robustness of our proposed protocol, the XGboost model was repeated for 1000 replicates. For each replicate, the training dataset was randomly chosen from the cumulative input dataset, and the above procedure was repeated. It should be emphasized that the goal of Schemes 1~3 is to establish a “structure–activity” relationship, whereas the goal of Scheme 4 is to establish a “structure–free energy” relationship.

Further, we employed the SHAP (Shapley additive explanations) method [26] to enhance the interpretability of the XGboost model [31]. SHAP is a method for explaining machine learning model predictions by providing an explanation of the contribution of each feature to a prediction. Specifically, SHAP values are based on the Shapley value concept from cooperative game theory, measuring the contribution of each feature when multiple features collaborate. SHAP values provide the following information:

(1) Feature importance: SHAP values help us understand the importance of each feature for the model’s predictions. Positive SHAP values indicate a positive impact on the prediction, while negative values indicate a negative impact.
(2) Explaining model predictions: By summing up the SHAP values of each feature, we can explain why the model made a specific prediction. This helps identify which features drive or suppress a particular prediction.

SHAP values offer an intuitive way to explain the predictions of complex machine learning models, helping users gain a better understanding of how the model operates, detect feature importance, uncover underlying data patterns, and identify critical features in the model’s predictions. This approach allows us to develop a holistic understanding of how individual structural features of SHP2 affect the model’s decision-making process. Consequently, it enhances our capacity to interpret the model’s classifications of various SHP2 conformation states.

## 4. Conclusions

This study deepens the understanding of the allosteric mechanism of SHP2 and the action of its inhibitors through equilibrium molecular dynamics simulations, meta-dynamics simulations, and the application of machine learning algorithms. Below is a comprehensive summary of the results obtained:

1. Thermodynamic interaction analysis: Equilibrium molecular dynamics simulations revealed the key residues and interactions between the inhibitor and SHP2, as well as between the N-SH2 and PTP domains. Hydrogen bonds and hydrophobic interactions between the inhibitor and residues such as Arg111, Phe113, Thr219, Glu250, Thr253, Leu254, Gln257, and Pro491 significantly enhance the stability of the inhibitor binding at the allosteric site of SHP2. The application of machine learning algorithms further confirmed the structural features, interaction fingerprints, and contact residue pairs most relevant to experimental inhibitory activity, particularly the hydrogen bonds between the inhibitor and Glu250, Arg111, and Leu216, as well as hydrophobic interactions with Gln257 and Phe113, which are crucial for enhancing experimental inhibitory activity.
2. Kinetic analysis of SHP2 conformational changes: Meta-dynamics simulations revealed that the transition (activation) process of the SHP2 protein from inactive to active conformations follows two distinct pathways. The binding of the inhibitor significantly increases the free energy barriers of both pathways, indicating that the inhibitor has a pronounced inhibitory effect on the kinetic process of SHP2 conformational change (activation). The application of machine learning algorithms further confirmed the interactions between specific residue pairs in the SHP2 protein: (1) Residues such as “104CYS-192ASP”, “71PHE-281ASN”, “131LYS-256GLN”, “218THR-229ARG”, and “114HIE-217ASN”, “23ARG-187PHE” contribute to enhancing the stability of the inactive conformation. (2) Residues such as “195GLU-317LYS”, “217ASN-492LYS”, “198LYS-315GLU”, and “109SER-232GLU” drive the allosteric (activation) process. (3) Residues such as “1MET-222ASN”, “58ASN-506GLN”, “4ARG-222ASN”, “61ASP-465ARG”, “3SER-101PRO”, and “63TYR-522ILE” are associated with the mechanism of action of the inhibitor.
3. Implications for drug design: This study provides significant insights for designing potent allosteric inhibitors of SHP2, particularly by enhancing interactions between specific residue pairs of the SHP2 protein to inhibit the allosteric process. This has potential clinical applications in developing novel anticancer drugs and addressing issues of drug resistance.

In summary, our research not only deepens the understanding of the allosteric mechanism of SHP2 protein but also provides important information for the design and development of the next generation of SHP2 allosteric inhibitors.

## Figures and Tables

**Table 1:** Kinetic data of apo-SHP2 and allosteric inhibitor-bound SHP2 during its conformational changes.

System	ΔG_exp_^a^	Δ$G_{\mathrm{active}}^{\neq }$ ^b^	Δ$G_{\mathrm{inactive}}^{\neq }$ ^b^	k_active_ ^c^	k_inactive_ ^c^	K_a′_ or K_a_^d^	K_i′_^e^	1/K_i′_K_a′_
apo-SHP2	/	70.28	67.00	1.44 × 10^−12^	5.14 × 10^−12^	2.80 × 10^−1^	/	/
RMC-4550	−16.67	131.90	29.03	5.95 × 10^−23^	1.28 × 10^−5^	4.64 × 10^−18^	9.00 × 10^−35^	2.40 × 10^51^
IACS-13909	−10.71	126.81	20.05	4.29 × 10^−22^	4.19 × 10^−4^	1.03 × 10^−18^	1.07 × 10^−29^	9.09 × 10^46^
SHP389	−8.57	99.19	31.74	1.93 × 10^−17^	4.48 × 10^−6^	4.31 × 10^−12^	1.84 × 10^−34^	1.26 × 10^45^
SHP099	−6.85	126.93	82.78	4.09 × 10^−22^	1.13 × 10^−14^	3.63 × 10^−8^	1.05 × 10^−30^	2.62 × 10^37^
Compound **7**	−4.41	120.76	111.03	4.48 × 10^−21^	1.96 × 10^−19^	2.29 × 10^−2^	5.14 × 10^−26^	8.51 × 10^26^

^a^ ΔG_exp_ = RT × ln(IC50), where IC50 is expressed in μM and ΔG_exp_ is expressed in kJ/mol. ^b^ Δ$G_{\mathrm{active}}^{\neq }$ and Δ$G_{\mathrm{inactive}}^{\neq }$ (in kJ/mol) represent the free energy barriers from inactive to active states and from active to inactive states, respectively. ^c^ The activation rate k_active_ and inactivation rate k_inactive_ of SHP2 are expressed in s^−1^. ^d^ The equilibrium constant K_a′_ between the active and inactive states is dimensionless. ^e^ The thermodynamic properties of inhibitor binding K_i′_ is expressed in mol/L.

## Data Availability

The data presented in this study are available upon request from the corresponding author.

## Supplementary Materials

The following supporting information can be downloaded at: https://www.mdpi.com/article/10.3390/molecules30010014/s1.
