# Supplementary material for: Study on SHP2 Conformational Transition and Structural Characterization of Its High-Potency Allosteric Inhibitors by Molecular Dynamics Simulations Combined with Machine Learning
Source: Molecules. 2024 Dec 24;30(1):14. doi: 10.3390/molecules30010014 (PMC11721961; doi:10.3390/molecules30010014)
Supplement: Supplementary file 1 [file molecules-30-00014-s001.zip › molecules-3309166-supplementary.pdf]

Supporting Information for

Study on SHP2 Conformational Transition and Structural Characterization of Its High-Potency Allosteric Inhibitors by Molecular Dynamics Simulations Combined with Machine Learning

Baerlike Wujieti, Mingtian Hao, Erxia Liu, Luqi Zhou, Huanchao Wang, Yu Zhang, Wei Cui * and Bozhen Chen *

School of Chemical Sciences, University of Chinese Academy of Sciences, No. 19A, Yuquan Road, Beijing 100049, China; wujietibaerlike21@mails.ucas.ac.cn (B.W.); haomingtian19@mails.ucas.ac.cn (M.H.); liuerxia21@mails.ucas.ac.cn (E.L.); zhouluqi22@mails.ucas.ac.cn (L.Z.); wanghuanchao22@mails.ucas.ac.cn (H.W.); zhangyu239@mails.ucas.ac.cn (Y.Z.)

* Correspondence: cuiwei@ucas.ac.cn (W.C.); bozhenchen@hotmail.com (B.C.)

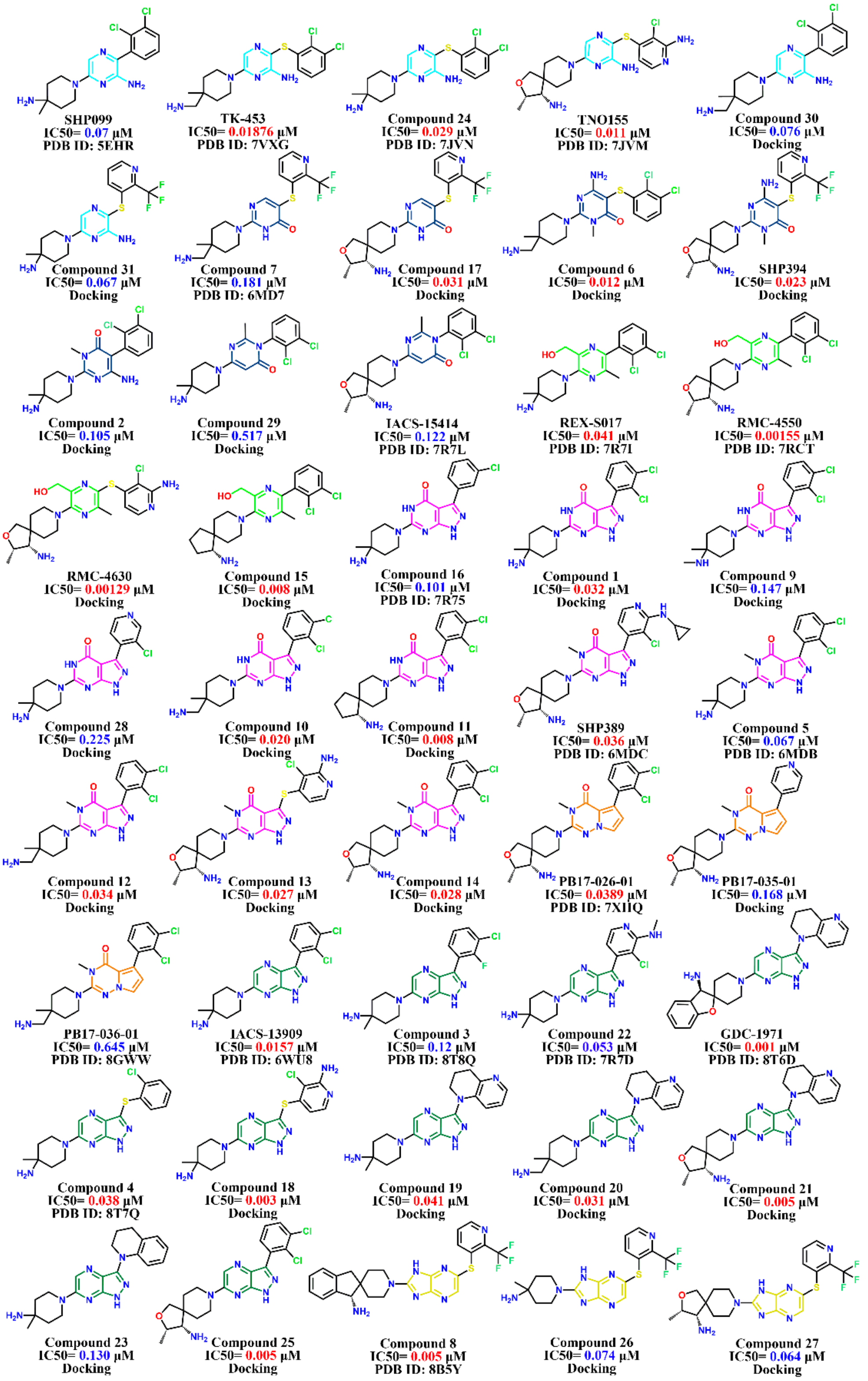

Figure S1 Two-dimensional structures, IC50 values, and co-crystal protein ID numbers of all allosteric inhibitors.

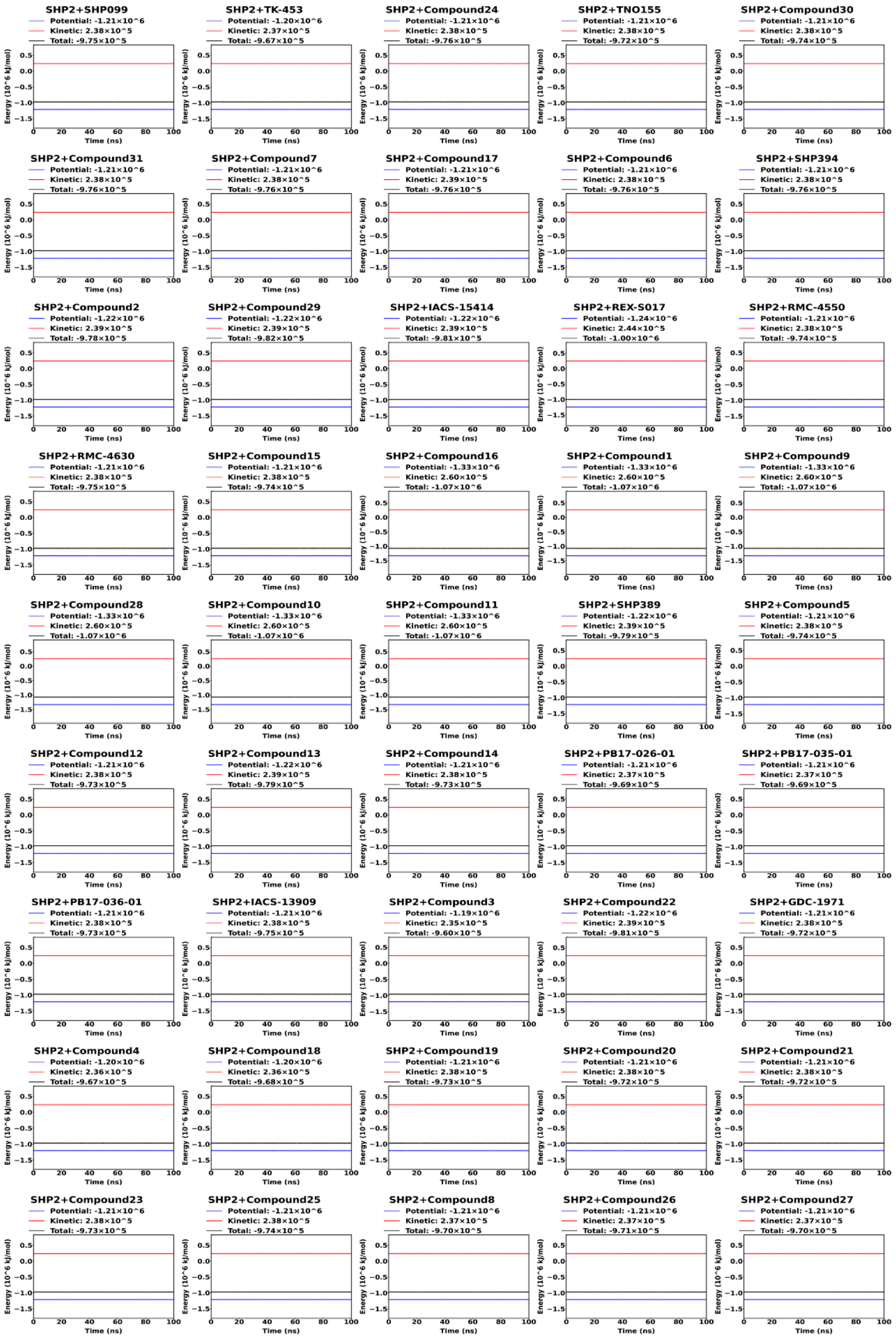

Figure S2 Kinetic, potential, and total energy change curves of the allosteric inhibitor-SHP2 complexes along equilibrium molecular dynamics simulation trajectories.

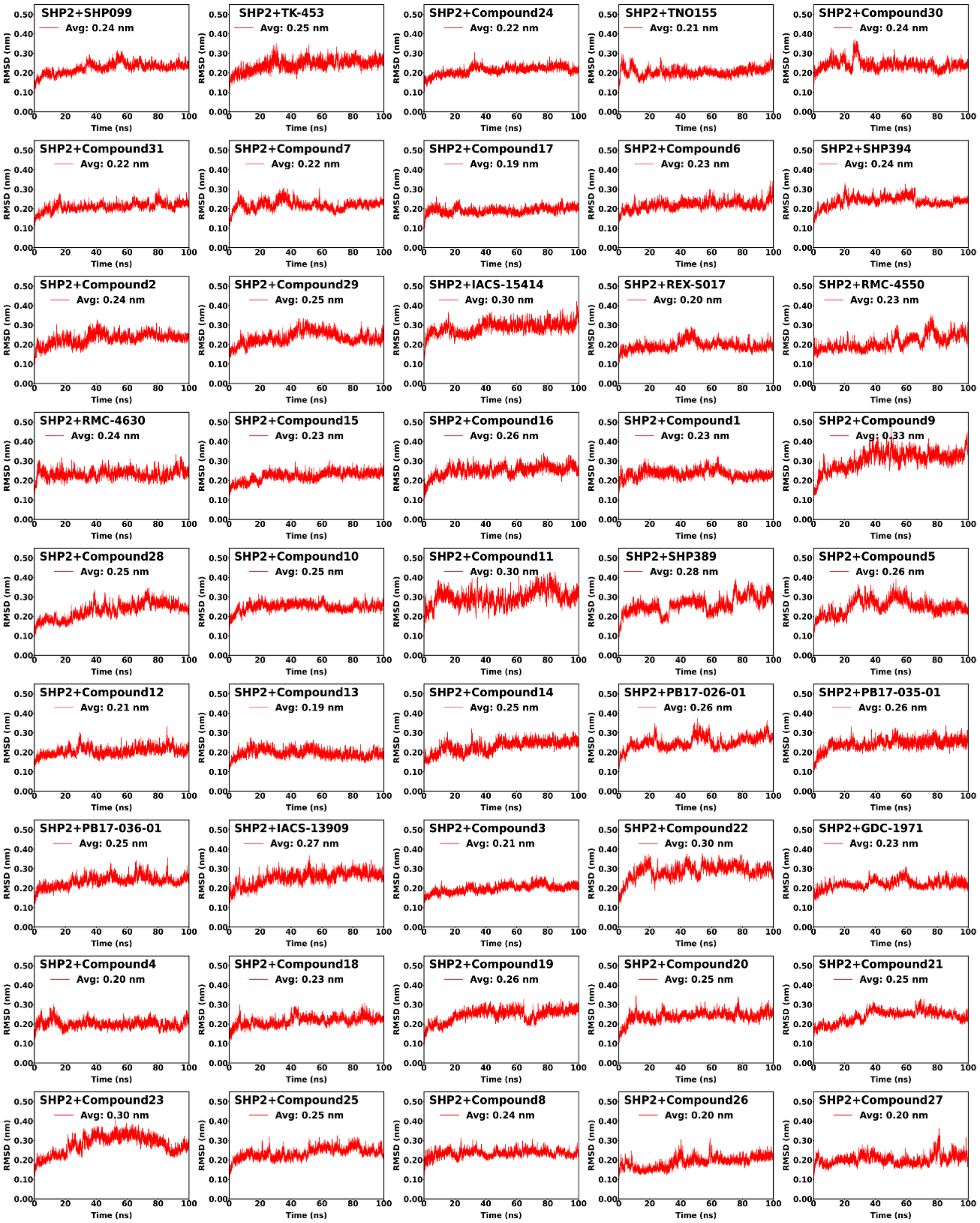

Figure S3 The RMSD value curves of the allosteric inhibitor-SHP2 complexes along the equilibrium molecular dynamics simulation trajectory.

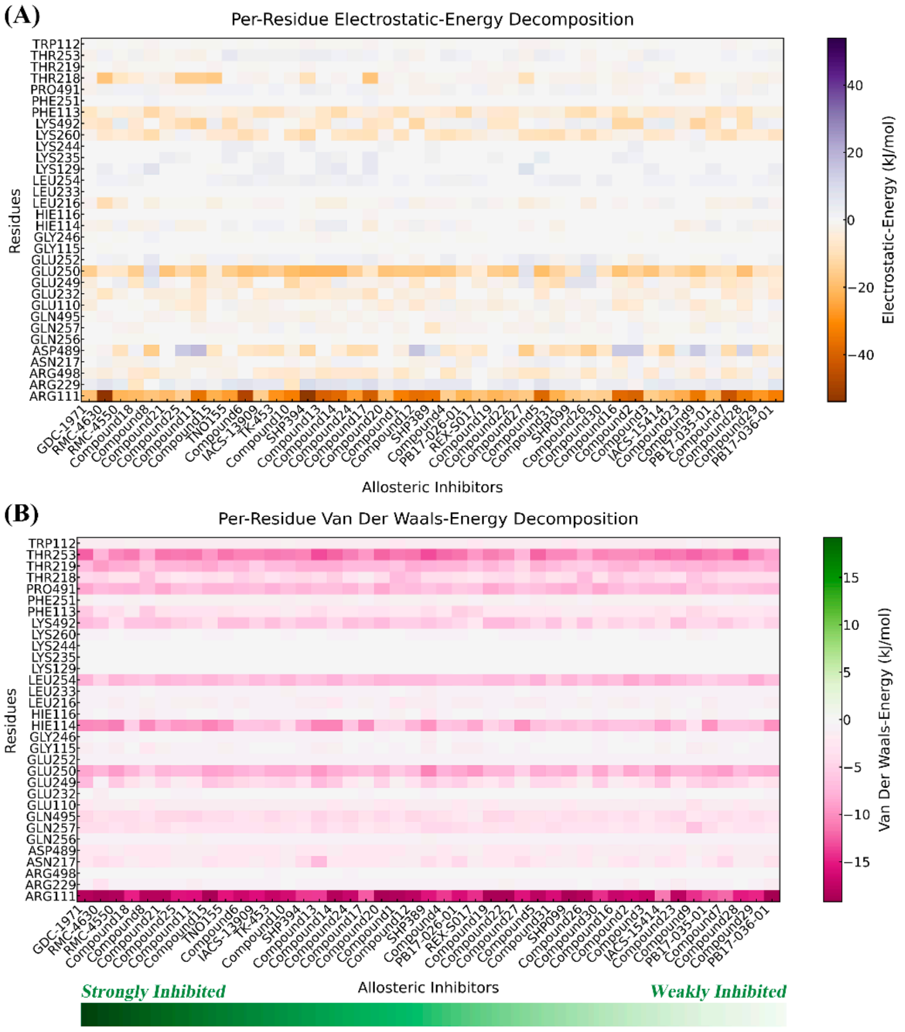

Figure S4 Interaction energies of electrostatic (A) and van der Waals (B) interactions between allosteric inhibitors and SHP2 residues.

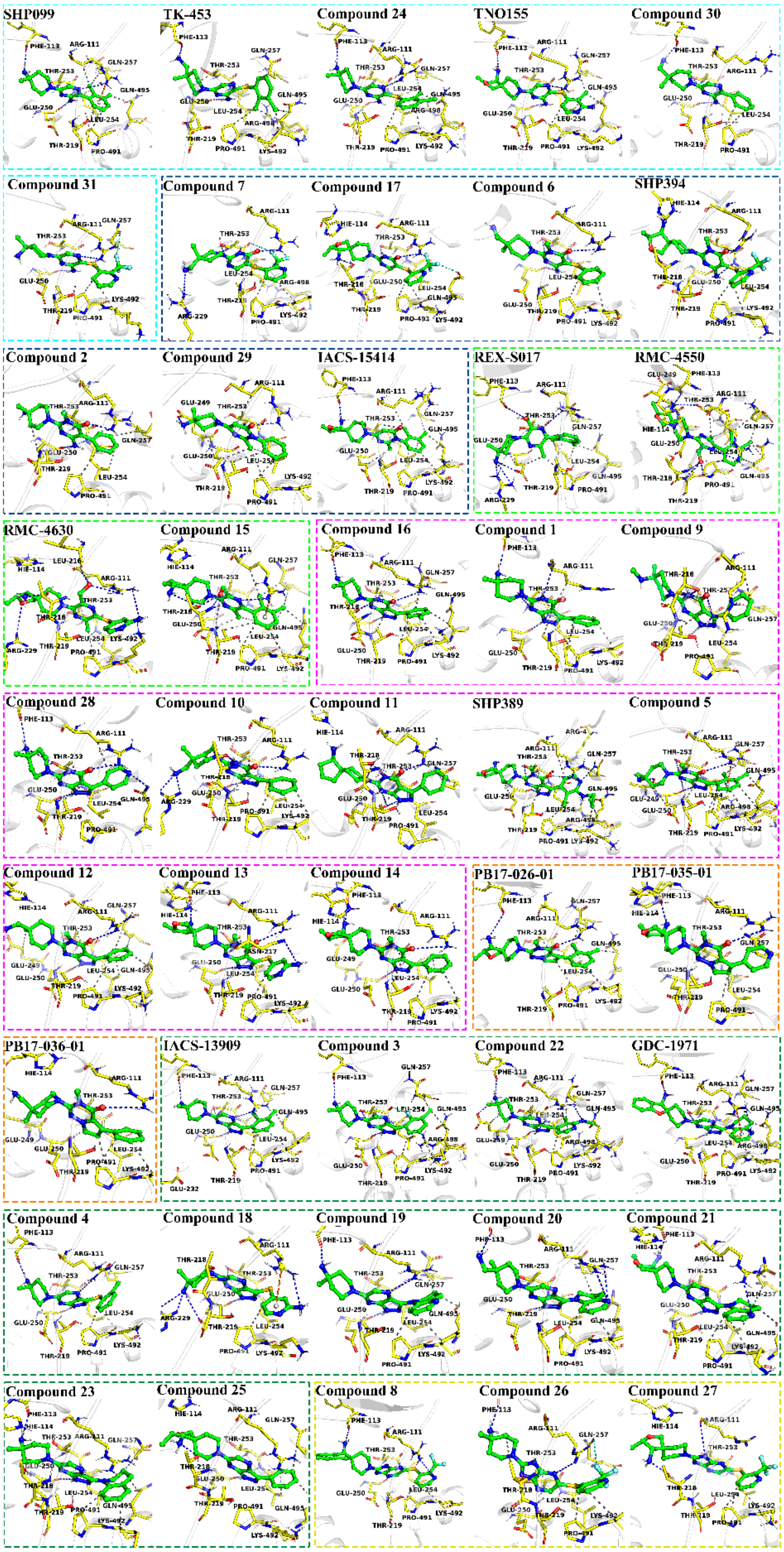

Figure S5 Interaction models of various allosteric inhibitors with SHP2.

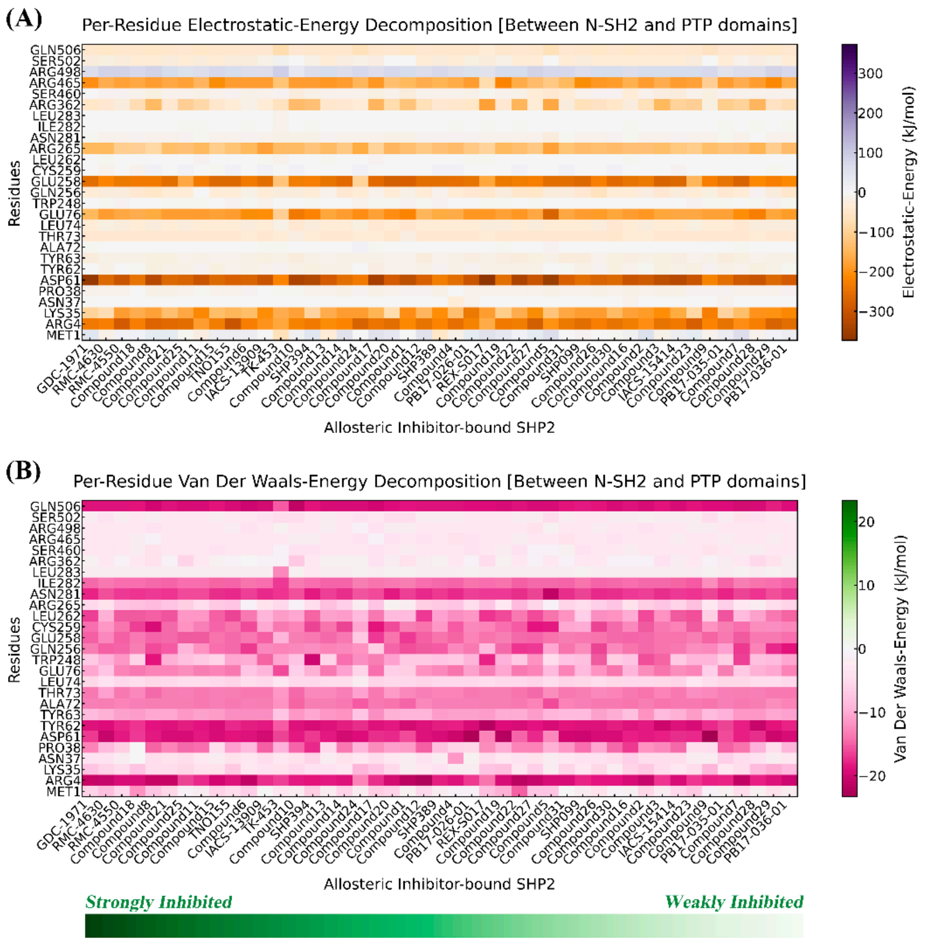

Figure S6 Interaction energies of electrostatic (A) and van der Waals (B) interactions between each residue pair of N-SH2/PTP structural domains.

$$V_{Max} = k[E_T]$$

$$V = k[E_{active}S]$$

$$[E_{active}] = \frac{K_M[E_{active}S]}{[S]}$$

$$[E_{inactive}] = \frac{[E_{active}]}{K_a} = \frac{K_M[E_{active}S]}{K_a[S]}$$

$$[E_{active}I] = \frac{[E_{active}][I]}{K_{i'}} = \frac{K_M[E_{active}S][I]}{K_{i'}[S]}$$

$$[E_{inactive}I] = \frac{[E_{active}I]}{K_{a'}} = \frac{K_M[E_{active}S][I]}{K_{a'} \quad K_{i'}[S]}$$

$$[E_{active}SI] = \frac{[E_{active}S][I]}{K_{i'}}$$

$$\frac{V_{Max}}{V} = \frac{k[E_T]}{k[E_{active}S]}$$

$$= \frac{[E_T]}{[E_{active}S]}$$

$$= \frac{[E_{active}] + [E_{inactive}] + [E_{active}I] + [E_{inactive}I] + [E_{active}SI] + [E_{active}S]}{[E_{active}S]}$$

$$= \frac{\frac{K_M[E_{active}S]}{[S]} + \frac{K_M[E_{active}S]}{K_a[S]} + \frac{K_M[E_{active}S][I]}{K_{i'}[S]} + \frac{K_M[E_{active}S][I]}{K_{a'} \quad K_{i'}[S]} + \frac{[E_{active}S][I]}{K_{i'}} + [E_{active}S]}{[E_{active}S]}$$

$$= \frac{K_M}{[S]} + \frac{K_M}{K_a[S]} + \frac{K_M[I]}{K_{i'}[S]} + \frac{K_M[I]}{K_{a'} \quad K_{i'}[S]} + \frac{[I]}{K_{i'}} + 1$$

$$\frac{1}{V} = \frac{K_M}{V_{max}}(1 + \frac{[I]}{K_{i'}} + \frac{1}{K_a} + \frac{[I]}{K_{i'} \quad K_{a'}})\frac{1}{[S]} + \frac{1}{V_{max}}(1 + \frac{[I]}{K_{i'}})$$

$$= \frac{K_M}{V_{max}}\frac{\alpha}{[S]} + \frac{\alpha'}{V_{max}}$$

$$\alpha = 1 + \frac{[I]}{K_{i'}} + \frac{1}{K_a} + \frac{[I]}{K_{i'} \quad K_{a'}}$$

$$\alpha' = 1 + \frac{[I]}{K_{i'}}$$

Figure S7 Derivation of the equation for enzyme-catalyzed reaction rate V versus substrate concentration [S].

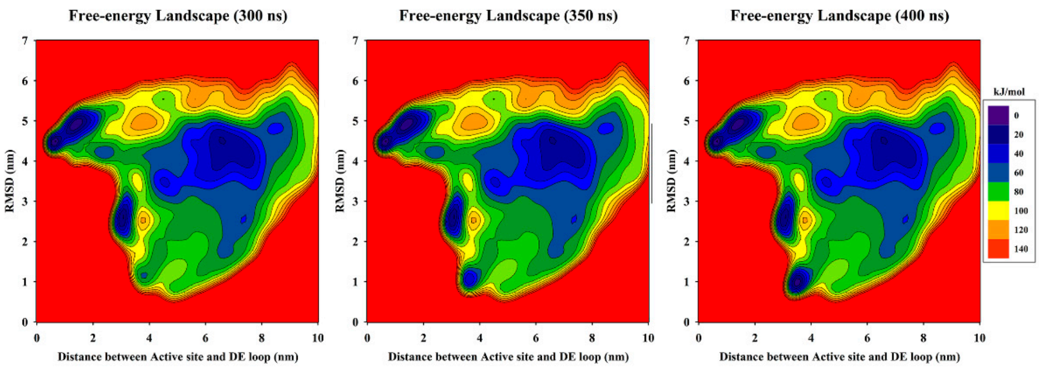

Figure S8 Free energy landscapes for meta-dynamics simulations of apo-SHP2 at 300 ns, 350 ns, and 400 ns.

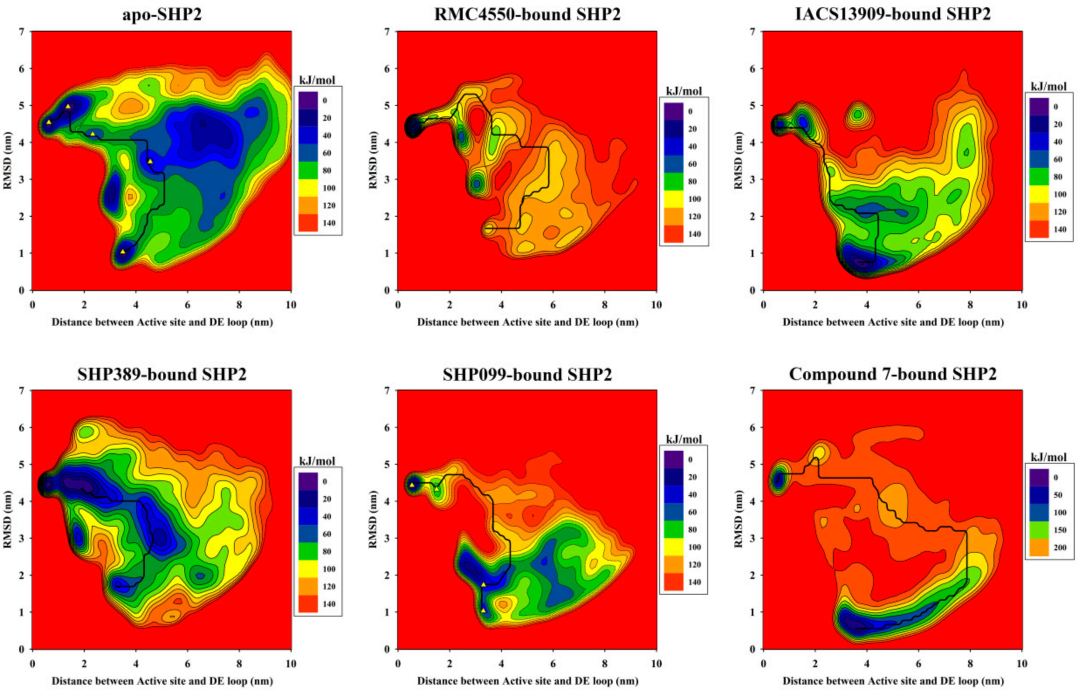

Figure S9 Free energy landscapes of the transformation process of apo-SHP2 and allosteric inhibitor-bound SHP2.

Table S1 Binding free energy of allosteric inhibitors with SHP2.

Ligand	ΔG_{exp} [a]	ΔE_{vdw} [b]	ΔE_{ele} [b]	ΔG_{GB} [b]	ΔG_{SA} [b]	ΔG_{bind} [b]
GDC-1971	-17.80	-273.25	-126.20	218.67	-28.51	-209.29
RMC-4630	-17.15	-234.41	-233.32	293.72	-28.13	-202.13
RMC-4550	-16.67	-248.26	-108.41	182.88	-28.34	-202.13
Compound18	-14.97	-212.43	-172.33	239.81	-23.57	-168.52
Compound21	-13.66	-247.05	-130.39	222.48	-26.12	-181.04
Compound25	-13.66	-216.32	-121.52	181.67	-22.56	-178.74
Compound8	-13.66	-234.03	-111.85	218.79	-26.75	-153.87
Compound15	-12.44	-239.93	-140.73	204.90	-27.29	-203.06
Compound11	-12.44	-219.05	-222.85	286.02	-23.69	-179.57
TNO155	-11.62	-236.84	-123.23	228.67	-26.29	-157.64
Compound6	-11.40	-221.60	-211.93	282.67	-24.91	-175.76
IACS-13909	-10.71	-208.92	-121.77	181.83	-23.15	-172.00
TK-453	-10.25	-225.87	-128.34	201.13	-25.74	-178.78
Compound10	-10.08	-211.13	-202.05	253.24	-23.69	-183.63
SHP394	-9.72	-224.61	-251.32	331.10	-27.12	-171.95
Compound13	-9.31	-265.93	-185.60	290.25	-27.96	-189.24
Compound14	-9.22	-260.36	-157.85	235.71	-28.34	-210.80
Compound24	-9.12	-217.83	-129.85	195.81	-24.53	-176.43
Compound20	-8.95	-245.37	-121.77	208.75	-25.58	-183.97
Compound17	-8.95	-197.99	-223.98	300.84	-24.82	-145.96
Compound1	-8.87	-219.25	-177.69	242.36	-23.40	-177.98
Compound12	-8.72	-230.35	-157.39	228.34	-24.28	-183.68
SHP389	-8.57	-268.06	-168.52	264.42	-28.13	-200.29
Compound4	-8.43	-217.58	-100.67	173.59	-23.78	-168.40
PB17-026-01	-8.37	-236.46	-131.31	195.44	-25.58	-197.91
Compound19	-8.23	-228.92	-134.16	211.30	-24.15	-175.97
REX-S017	-8.23	-205.15	-90.67	160.78	-23.82	-158.81
Compound22	-7.57	-224.32	-124.19	199.12	-24.19	-173.55
Compound27	-7.08	-200.42	-104.23	203.43	-24.57	-125.74
Compound5	-6.97	-230.35	-163.62	228.92	-25.24	-190.25
Compound31	-6.97	-207.37	-160.99	263.33	-26.08	-131.10
SHP099	-6.85	-210.63	-75.05	131.23	-23.52	-177.98
Compound26	-6.71	-199.79	-82.54	179.41	-24.28	-127.21
Compound30	-6.64	-209.92	-75.26	144.41	-23.78	-164.50
Compound16	-5.91	-205.78	-185.14	233.03	-22.48	-180.37

Compound2	-5.81	-214.44	-150.48	216.32	-24.03	-172.62
Compound3	-5.46	-210.67	-110.84	168.56	-23.65	-176.60
IACS-15414	-5.42	-225.41	-93.26	182.38	-25.12	-161.41
Compound23	-5.26	-225.70	-130.31	188.41	-24.70	-192.30
Compound9	-4.94	-219.88	-185.43	236.46	-22.27	-191.13
PB17-035-01	-4.60	-229.80	-117.62	207.79	-25.91	-165.59
Compound 7	-4.41	-208.25	-204.40	288.87	-26.37	-150.15
Compound 28	-3.84	-207.16	-166.68	247.01	-22.69	-149.52
Compound 29	-1.70	-201.30	-122.73	195.02	-22.65	-151.70
PB17-036-01	-1.13	-234.11	-132.86	210.51	-25.70	-182.17

[a] $\Delta G_{exp} = RT \times \ln(IC50)$, where IC50 is expressed in μM and ΔG_{exp} is expressed in kJ/mol. [b] Thermodynamic data such as ΔE_{vdw}, ΔE_{ele}, ΔG_{GB}, ΔG_{SA} and ΔG_{bind} are expressed in kJ/mol.

Table S2 Binding free energy between the N-SH2 and PTP domains.

System	ΔG_{exp} [a]	ΔE_{vdw} [b]	ΔE_{ele} [b]	ΔG_{GB} [b]	ΔG_{SA} [b]	ΔG_{bind} [b]
GDC-1971	-17.80	-549.94	-2871.43	3134.88	-82.00	-368.48
RMC-4630	-17.15	-525.94	-2713.68	3033.47	-74.29	-280.44
RMC-4550	-16.67	-527.40	-2969.56	3263.23	-74.86	-308.59
Compound18	-14.97	-507.36	-2592.59	2895.60	-73.25	-277.61
Compound8	-13.66	-586.16	-3035.37	3330.25	-82.67	-373.96
Compound21	-13.66	-559.55	-2948.03	3246.37	-79.46	-340.67
Compound25	-13.66	-518.14	-2902.03	3221.44	-75.82	-274.55
Compound11	-12.44	-520.86	-2946.20	3254.32	-77.00	-289.73
Compound15	-12.44	-528.46	-2697.72	3010.89	-75.66	-290.95
TNO155	-11.62	-551.75	-2918.39	3202.52	-77.10	-344.71
Compound6	-11.40	-516.45	-2765.44	3044.04	-72.58	-310.43
IACS-13909	-10.71	-518.28	-3065.60	3297.14	-75.36	-362.11
TK-453	-10.25	-499.59	-2554.37	2907.49	-68.92	-215.39
Compound10	-10.08	-522.19	-2998.83	3219.61	-76.34	-377.75
SHP394	-9.72	-546.91	-2782.21	3030.78	-77.31	-375.65
Compound13	-9.31	-523.64	-2963.22	3271.32	-76.83	-292.38
Compound14	-9.22	-522.89	-2770.12	3076.76	-72.89	-289.15
Compound24	-9.12	-487.88	-2841.04	3104.93	-73.35	-297.35
Compound17	-8.95	-555.75	-3004.07	3296.72	-77.59	-340.69
Compound20	-8.95	-520.66	-2827.74	3095.26	-74.08	-327.22
Compound1	-8.87	-506.85	-2843.60	3129.56	-74.48	-295.37
Compound12	-8.72	-534.01	-2751.47	3051.02	-76.54	-310.99
SHP389	-8.57	-514.33	-2907.01	3251.78	-73.86	-243.43
Compound4	-8.43	-535.00	-2743.54	3045.22	-77.76	-311.07
PB17-026-01	-8.37	-511.11	-2987.08	3236.87	-74.70	-336.04
REX-S017	-8.23	-548.69	-2780.75	3103.04	-79.48	-305.88
Compound19	-8.23	-570.79	-2784.42	3061.03	-83.14	-377.32
Compound22	-7.57	-556.02	-3022.44	3329.40	-79.99	-329.04
Compound27	-7.08	-587.66	-3118.03	3371.12	-86.67	-421.25
Compound5	-6.97	-502.87	-2891.96	3116.83	-72.65	-350.66
Compound31	-6.97	-518.27	-2856.71	3133.16	-77.36	-319.17
SHP099	-6.85	-494.39	-2937.16	3187.62	-73.24	-317.17
Compound26	-6.71	-566.36	-2980.46	3283.74	-80.50	-343.58
Compound30	-6.64	-524.28	-2872.31	3170.73	-76.73	-302.59
Compound16	-5.91	-535.36	-2916.94	3175.73	-80.11	-356.68
Compound2	-5.81	-553.08	-2869.29	3171.42	-79.29	-330.25
Compound3	-5.46	-539.55	-3228.93	3521.74	-79.19	-325.94
IACS-15414	-5.42	-528.10	-2942.62	3227.12	-78.92	-322.52
Compound23	-5.26	-523.23	-2651.50	2940.55	-76.23	-310.41
Compound9	-4.94	-509.61	-2781.39	3090.72	-73.18	-273.46
PB17-035-01	-4.60	-512.30	-2615.63	2914.80	-73.95	-287.08

Compound7	-4.41	-524.73	-2760.36	3040.10	-72.76	-317.74
Compound28	-3.84	-570.89	-3082.38	3371.20	-85.42	-367.48
Compound29	-1.70	-523.58	-3109.00	3390.65	-77.44	-319.37
PB17-036-01	-1.13	-556.18	-3137.79	3408.97	-82.51	-367.52
5EHR	/	-537.23	-3053.80	3364.33	-78.97	-305.67
6MD7	/	-505.87	-2569.78	2853.67	-69.52	-291.50
6MDC	/	-548.65	-2745.35	3027.53	-78.04	-344.50
6WU8	/	-534.46	-3407.44	3659.08	-80.78	-363.59
7RCT	/	-551.97	-3100.79	3386.08	-81.41	-348.10

[a] $\Delta G_{exp} = RT \times \ln(IC50)$, where IC50 is expressed in μM and ΔG_{exp} is expressed in kJ/mol. [b] Thermodynamic data such as ΔE_{vdw}, ΔE_{ele}, ΔG_{GB}, ΔG_{SA} and ΔG_{bind} are expressed in kJ/mol.

Table S3 The various types of machine learning regression models trained on equilibrium molecular dynamics simulation data and their evaluation results.

Regressor Mode		R^2	MSE	RMSE	MAE
Trajectory Analysis Data	XGBoost	0.66	0.00	0.03	0.02
	KNN	0.45	0.00	0.04	0.02
	LR	0.52	0.00	0.03	0.02
	RF	0.60	0.00	0.03	0.02
	SVM	0.49	0.00	0.04	0.02
	DT	0.51	0.00	0.03	0.02
Interaction Fingerprints	XGBoost	0.71	0.03	0.03	0.05
	KNN	0.41	0.05	0.09	-0.03
	LR	0.52	0.02	0.00	0.03
	RF	0.62	0.02	0.05	0.05
	SVM	0.46	0.01	-0.01	0.02
	DT	0.52	-0.03	0.06	0.06
Residue Contact Matrix	XGBoost	0.65	-0.03	0.01	0.02
	KNN	0.42	0.02	0.08	0.05
	LR	0.50	0.03	0.05	0.01
	RF	0.60	0.00	0.04	-0.02
	SVM	0.46	-0.03	-0.01	0.06
	DT	0.48	-0.03	0.02	0.02

Table S4 The various types of machine learning classification models trained on equilibrium molecular dynamics simulation data and their evaluation results.

Classification Mode		Accuracy	Precision	Recall	F1
Trajectory Analysis Data	XGBoost	0.84	0.84	0.83	0.83
	KNN	0.78	0.78	0.78	0.78
	LR	0.83	0.83	0.83	0.82
	RF	0.81	0.81	0.81	0.81
	SVM	0.65	0.65	0.65	0.65
	DT	0.77	0.77	0.77	0.77
Interaction Fingerprints	XGBoost	0.84	0.87	0.83	0.86
	KNN	0.78	0.81	0.81	0.79
	LR	0.81	0.85	0.79	0.83
	RF	0.80	0.86	0.79	0.76
	SVM	0.69	0.63	0.60	0.63
	DT	0.76	0.76	0.76	0.77
Residue Contact Matrix	XGBoost	0.82	0.80	0.78	0.85
	KNN	0.76	0.76	0.82	0.80
	LR	0.82	0.82	0.81	0.78
	RF	0.78	0.83	0.76	0.77
	SVM	0.61	0.69	0.67	0.64
	DT	0.79	0.80	0.76	0.77

Table S5 The various types of machine learning regression models trained on residue contact matrix data from meta-dynamics simulations and their evaluation results.

Regressor Mode			R^2	MSE	RMSE	MAE
apo-SHP2	Residue Contact Matrix	XGBoost	0.93	56.09	7.49	5.10
		KNN	0.85	78.36	8.86	6.20
		LR	0.78	98.23	9.91	7.50
		RF	0.91	64.57	8.03	5.68
		SVM	0.80	89.12	9.45	6.99
		DT	0.87	74.99	8.65	6.05
Inhibitor-bound SHP2	Residue Contact Matrix	XGBoost	0.97	60.22	7.76	5.14
		KNN	0.91	75.12	8.66	6.32
		LR	0.87	92.54	9.62	7.23
		RF	0.96	68.43	8.27	5.99
		SVM	0.93	82.35	9.07	6.54
		DT	0.90	88.77	9.42	7.01
